# Is the Excessive Use of Microblogs an Internet Addiction? Developing a Scale for Assessing the Excessive Use of Microblogs in Chinese College Students

**DOI:** 10.1371/journal.pone.0110960

**Published:** 2014-11-18

**Authors:** Juan Hou, Zhichao Huang, Hongxia Li, Mengqiu Liu, Wei Zhang, Ning Ma, Lizhuang Yang, Feng Gu, Ying Liu, Shenghua Jin, Xiaochu Zhang

**Affiliations:** 1 Department of Philosophy, Anhui University, Hefei, Anhui, P.R. China; 2 CAS Key Laboratory of Brain Function and Disease, and School of Life Science, University of Science and Technology of China, Hefei, Anhui, P.R. China; 3 Department of Psychology, Beijing Normal University, Beijing, P.R. China; 4 Anhui Provincial Hospital (affiliated to Anhui Medical University), Hefei, Anhui, China, P.R. China; 5 School of Humanities and Social Science, University of Science and Technology of China, Hefei, Anhui, P.R. China; University of Pennsylvania, United States of America

## Abstract

More and more college students are using microblogs, with some excessive users demonstrating addiction-like symptoms. However, there is currently no published scale available for use in assessing excessive use of these microblogs, a significant impediment to advancing this area of research. We collected data from 3,047 college students in China and developed a Microblog Excessive Use Scale (MEUS) for Chinese college students, comparing it with criteria used for assessing Internet addiction. Our diagnostic scale featured three factors, two of which–“withdrawal and health problem” and “time management and performance”–are already included in Internet addiction assessment scales. The third factor, “social comfort,” does not appear in Internet addiction assessment scales. Our study found that females have significantly higher MEUS scores than males, and that total MEUS scores positively correlated with scores from “self-disclosure” and “real social interaction” scales. These findings differ from results obtained in previous investigations into Internet addiction. Our results indicate that some characteristics of the excessive use of microblogs are different to those of Internet addiction, suggesting that microblog overuse may not correspond exactly to the state of Internet addiction.

## Introduction

Recent times have witnessed the growth of Internet-based communication technologies; creating a new field of academic study focusing on social networks [1]. Social networking sites like MySpace and Facebook have grown quickly and have had significant impact on young people [2]. However, the emergence of microblogging sites like Twitter has shifted online communication and interaction trends. Many people have embraced this new form of communication in that nearly 40% of social networking sites users have migrated to microblogs [3]. Microblogs are particularly popular in the United States (Twitter) and in China (Weibo; in particular, Sina Weibo) [4]. For instance, according to Twitter’s 2013 Initial Public Offerings (IPO) filing [5], [6], the company registered a growth rate of 1,400% per year, had more than 215 million registered accounts, over 100 million daily active users, and boastes of “approximately 500 million Tweets every day”. In 2012, the number of microblog users in China surpassed 300 million, making China the country with highest number of microblog users in the world [7]. Most of these microblog users are college students (72.3%) [8] and microblogging is now considered an indispensable part of a college student’s life.

Due to the increase in number of microblog users, microblogging has attracted scholarship from different academic disciplines. Mainly, scholars believe that microblog use or overuse may present symptoms of behavioral addiction (e.g., [1]). Already, many studies have been conducted on Internet addiction (e.g., [9], [10]), as well as on people’s engagement in other Internet activities including online gaming [11], [12], social networking [13], mobile emailing [14], instant messaging [15], and online auctioning [13]. Most researchers believe that these Internet-related behaviors may be some form of Internet addiction, and have hence measured them against the central thinking in addiction studies [16]. However, in contrast to other forms of addiction like drug addiction and addictive behaviors such as gambling, Internet-related behaviors are not homogenous and as such different applications and use of the Internet may exhibit different addictive characteristics [17], [18]. Only few studies have focused on the use of microblogs and for these few studies, researchers have used Internet addiction diagnostic criteria to assess the excessive use of microblogs [19]. For example, Ma (2012) states that individuals who use microblogs for more than six hours every day and whose behavior meets the diagnostic criterion of Internet addiction are excessive users of microblogs [20].

At present, several Internet addiction diagnostic criteria have been published (e.g., [21]–[28]). Although the standard for Internet addiction is not exactly the same across all of them, each includes the basic characteristics of Internet addiction namely: impulse, loss of control, and insistence of negative impact behaviors [29]. Researchers have also determined that Internet addiction can lead to a variety of negative consequences such as decreased involvement in real-life communities, worsening of academic performance, and an increase in relationship problems [30]. Young’s 20-item Internet Addiction Test (IAT) [31] was the first published diagnostic scale; we utilized it in our study to measure Internet addiction. Chang and Law’s confirmatory study [32] identified three factors from Young’s IAT: (1) “withdrawal and social problems,” (2) “time management and performance,” and (3) “reality substitute.”

As a behavior related to Internet-based applications, excessive use of microblogs comprises a few of the basic characteristics of Internet addiction associated with social networking. Excessive use of this kind of online media can lead to, for example, misuse, dependence, and addiction [33]. That is, the excessive use of microblogs may bring about symptoms or outcomes similar to those seen in Internet addiction [34], including withdrawal effects and time-management problems (e.g., diminished quality and/or quantity of sleep time due to all-day use of microblogs). This is consistent with outcomes associated with the problematic or compulsive use of Facebook, which may contribute to the severity of symptoms associated with Internet addiction [35], such as poor sleep quality [36]. However, in contrast to another characteristic of Internet addiction, that of using the Internet for escapism [31], [32], [37], the primary reason for using microblogs is self-presentation [38], [39]; that is, discussing shared-interest topics or hot news to broaden one’s social networking size [40].

Therefore, there are similarities as well as differences between excessive use of microblogs and Internet addiction. As problematic use of microblogs may be a form of Internet addiction, and since the overall use of microblogs is increasing rapidly, there is need for a psychometrically sound procedure for assessing excessive use of microblogs. In the present study, we developed a scale, the Microblog Excessive Use Scale (MEUS) modeled after Young’s (1998) 20-item IAT [31], and reviewed its psychometric properties. We also explored the characteristics of microblog overuse among several samples of college students in China, and assessed the similarities and differences between IAT and MEUS.

## Methods and Materials

### Participants

The study targeted college students in China. The total number of participants was 3,047. Participants were recruited in and divided into five phases/samples. Participants in Sample 3 were recruited online while participants in the other samples were recruited from five colleges in China. The amount and quality of information collected varied across these samples (see Table 1). Participants were selected on the premise that they had had at least one microblog account and were college students. Initially, before the comprehensive study, 10 college students (5 males and 5 females) were recruited for an index test.

**Table 1:** Summary of the demographic data of participants.

Sample	*N*	Male (%)	Age (*M* ± *SD*)	*N* of collection (rate of availability)	Geographic regions
**Sample 1**	408	52.5	21.2±2.0	480 (85.0%)	Eastern China
**Sample 2**	200	38.5	22.9±1.7	240 (83.3%)	Eastern China
**Sample 3**	953	36.4	22.0±3.0	953 (100%)	all of China’s regions except Eastern China
**Sample 4**	381	75.6	18.2±1.5	480 (79.4%)	Eastern China
**Sample 5**	996	76.6	18.3±1.8	1280 (77.9%)	Eastern China
**Norm**	1949	57.0	20.1±3.1	2233 (87.3%)	all of China’s regions

*Note*. Norm comprises sample 3 and sample 5.

### Ethics Statement

Each participant to the study provided written informed consent after receiving an explanation of the study’s purpose and procedure. The study was approved by the Human Research Ethics Committee of the University of Science and Technology of China (USTC) according to the principles expressed in the Declaration of Helsinki. Participants were undergraduate and postgraduate students. We did not obtain informed consent from the next of kin, caretakers, or guardians on behalf of the minors/children enrolled in our study. Informed consent was obtained in written form from all participants.

We did not obtain informed consent from guardians of participants whose age was under 18. These young college students were considered to have comparable intelligence and ability to adult students, and able to take charge of their behaviors. According to the General principles of the Civil Law of the People’s Republic of China; “A minor aged 10 or over shall be a person with limited capacity for civil conduct and may engage in civil activities appropriate to his age and intellect; in other civil activities, he shall be represented by his agent *ad litem* or participate with the consent of his agent *ad litem*” (Article 12, Chapter II).

### Data Collection

Planning for data collection took place before the index test. The data in samples were collected a month after the index test in two-month intervals. The collection rate and availability rate is shown in Table 1. Participants who failed to complete the questionnaire were excluded from the analyses. In addition to our MEUS, Sample 4 also completed the IAT developed by Young [31], and Sample 5 also completed the Jourard Self-Disclosure Questionnaire [41] and our revised Social Interaction Scale [42].

### Measures

#### Demographic data

Each participant answered five questions concerning their gender, age, academic background, and other relevant information; see Supporting Information S1.

#### Characteristics of microblog use

Characteristics of microblog use by the participants were examined using eight questions (see Supporting Information S1). This section of our study examined participants’ primary behavior in relation to microblog use, including their perception on the importance of microblogging, their perceived dependency on it, first time using it, average time spent microblogging each day, frequency of browsing microblogs, and the average time spent on microblogs in a single session.

#### MEUS

Our questionnaire comprised 27 statements. The first 20 were modeled after Young’s IAT [31]–we simply replaced “Internet” with “microblog.” In addition, we featured further seven questions, which were based on responses obtained through our microblog interviews (see Supporting Information S2). For every statement (item) on our questionnaire, participants reported the extent to which that statement was true for them using a rating scale ranging from “1” (never) to “6” (always).

#### IAT

As noted earlier, the IAT is a 20-item, self-report measure of excessive Internet use [31]. This widely applied measure showed adequate internal consistency (α = .950) in our study. In the test, participants rated statements (items) on a six-point scale from “1” (does not apply) to “6” (always). Item scores were added together to obtain an overall score, with higher total scores indicating greater addiction-like symptoms.

#### Social Interaction Scale

We used the Social Interaction Scale, as revised by Yan [42], first developed by Chen and colleagues [43]; adapting it for the present study, we replaced the term “Internet” with “microblog.” The Social Interaction Scale comprised two subscales-a Real Social Interaction Scale and a Microblog Social Interaction Scale. Each subscale featured 12 statements. The Real Social Interaction Scale measured interpersonal communication with classmates, friends, parents, and other people in real life; for example, “parental intimacy,” “intimacy with friends,” and “informational disclosure with friends.” The Microblog Social Interaction Scale measured an individual’s interaction with others on Microblog and included factors such as “intimacy revealed” and “informational disclosure”. Participants rated each of the Social Interaction Scale’s 24 statements on a four-point scale ranging from “1” (never) to “4” (always). Individual item scores were added together to obtain a total score, with higher total scores indicating greater real or microblog interpersonal communication. The two scales showed good internal consistency (α = .845, α = .909) in our samples.

#### Self-Disclosure Questionnaire

We employed the Jourard Self-Disclosure Questionnaire [41] in our study to measure the extent to which individuals disclose information in real life. The Jourard Self-Disclosure Questionnaire has 60 statements (items) presented across six sections, with each section featuring 10 items. The questionnaire measures the degree or extent of information exchange with parents or friends on attitudes/opinions, interests/hobbies, study/work, money, personality, body, and so on. Participants rated each of the 60 items on a four-point scale: “0” means “Have told the other person nothing about this aspect of me”; “1” means “Have talked in general terms about this. The other person has only a general idea about this aspect of me”; “2” means “Have talked in full and complete detail about this item to the other person. They know me fully in this respect and could describe me accurately”; and “X” means “Have lied or misrepresented myself to the other person so that they have a false picture of me.” (X was given the value of 0). Item scores were added together to obtain a total score, with higher total scores indicating a greater degree of disclosure in real life. This scale showed good internal consistency (α = 0.964) in our study.

### Statistical analysis

We informed the participants that the purpose of this study was to examine the use of Microblogs. Five people read the index test and were masked to the results of the other tests. There were no adverse events from performing the index test.

Data were analyzed using the statistics software package SPSS 18.0 and exploratory factor analysis (EFA) was performed. The data from Sample 1 and Sample 2 were used for item discrimination analysis and factor analysis to determine the number of items. The data from Sample 3, collected online, were used to confirm the factor structure. Confirmatory factor analysis (CFA) was performed using Amos 7.0 software. The model fit indices indicated adequate model fit; the comparative fit index (CFI) was .953; the normed fit index (NFI) was .944; the relative fit index (RFI) was .916; and the incremental fit index (IFI) was .954. The root mean square error of approximation (RMSEA) was .069. According to Steiger (2007) [44], a stringent upper limit of 0.07 for RMSEA seems to be the consensus amongst authorities. To compare and identify differences between the excessive use of microblogs and Internet addiction, we asked participants in Sample 4 to complete both the MEUS and the IAT. To determine which psychological characteristics are associated with microblog use, we asked participants in Sample 5 to complete the Social Interaction Scale and the Self-Disclosure Questionnaire as well as our MEUS. Finally, we combined Sample 3 and Sample 5 data in order to calculate the norm of the MEUS, as well as to identify a standard for the assessment of excessive use of microblogs among college students. We calculated correlations between the scales using Spearman’s rho (Spearman’s rank correlation coefficient). To test the differences of different levels in the total score, we used the Kruskal-Wallis test (Kruskal-Wallis one-way analysis of variance) and the Mann-Whitney *U* test. The significance level in this study was *p*<.01.

## Results

The study was conducted from May 2012 to October 2013.

### Psychometric Properties of the MEUS–Sample 1

#### Item discrimination analysis

We computed the difference of each item in our 27-item questionnaire between two groups using the scores of two statements (the average numbers of Tweets browsed per day and the frequency of browsing Tweets per day). With reference to previous studies [45], [46], the high-scoring group included the top 27% participants and the low-scoring group included the bottom 27% participants. Results showed that all items except for item 3 had significant differences (*p*<.01), suggesting that this scale could identify the reaction degree of the different subjects.

#### EFA

Our 27-item MEUS showed good internal consistency (α = .946). We used EFA followed by oblique rotation to explore the underlying structure of these items. Factor loadings equal to and above 0.50 were considered indicative of items belonging to a specific factor. We extracted three factors, which accounted for 60.08% of total variance. After EFA, 23 items remained from the original 27. The specific item loadings on these three factors are listed in Table 2.

**Table 2:** Factor loadings from EFA of the 27-item MEUS.^a^

Items	Factor 1(withdrawal and health problem)	Factor 2(social comfort)	Factor 3(time management and performance)	Correcteditem–total correlation
23. How often do you sleep less than four hours a day becauseof using microblogs?	0.842	0.082	0.038	0.626^**^
21. How is your health affected by using microblogs?	0.821	0.025	−0.057	0.690^**^
20. How often do you feel depressed, moody, or nervous whenyou are off microblogs?	0.789	0.112	0.015	0.696^**^
22. How often do you continue to use microblogs, even thoughthis use has resulted in continuous or recurrent physical, social, or psychological problems?	0.781	0.105	0.101	0.637^**^
19. How often do you choose to spend more time on microblogsover going out with others?	0.748	0.145	−0.050	0.672^**^
13. How often do you snap, yell, or act annoyed if someonebothers you while you are using microblogs?	0.691	0.008	−0.074	0.686^**^
3. How often do you prefer the excitement of microblogs tointimacy with your partner?	0.682	−0.139	−0.133	0.430^**^
17. How often do you try to cut down the amount of timeyou spend on microblogs but fail to do so?	0.578	0.027	−0.340	0.726^**^
15. How often do you feel preoccupied with microblogs when off-line,or fantasize about being on a microblog?	0.566	−0.048	−0.209	0.654^**^
18. How often do you try to hide how long you have been using microblogs?	0.538	0.177	−0.036	0.651^**^
5. How often do others in your life complain to you about the amountof time you spend on microblogs?	0.526	0.040	−0.331	0.546^**^
12. How often do you feel life is empty and boring whenyou are unable to use microblogs?	0.393	0.216	−0.298	0.733^**^
25. How often do you feel excited when others Retweet your Tweetsor comment on your microblogs?	−0.255	0.744	−0.251	0.564^**^
24. How often do you change your mood because of a change in thenumber of fans?	0.143	0.654	−0.106	0.632^**^
27. How often do you get more followers by increasing the number that you are following?	0.330	0.642	0.130	0.579^**^
26. How often would you try to increase your followersunconsciously by all means?	0.488	0.541	0.137	0.581^**^
11. How often do you find yourself anticipating when you will usemicroblogs again?	0.051	0.535	0.094	0.589^**^
1. How often do you find yourself spending more time on microblogsthan you anticipating?	−0.234	0.048	−0.774	0.512^**^
2. How often do you neglect household chores to spend moretime on microblogs?	0.243	−0.010	−0.667	0.542^**^
8. How often does your job performance or productivity sufferbecause of your use of microblogs?	0.236	0.114	−0.608	0.430^**^
7. How often do you check your microblog before something else that you need to do?	0.077	0.287	−0.578	0.627^**^
16. How often do you find yourself saying “just a few more minutes”when using microblogs?	0.234	0.025	−0.552	0.643^**^
14. How often do you lose sleep due to late-night log-ins?	0.268	−0.085	−0.527	0.621^**^
6. How often do your grades or schoolwork suffer because of theamount of time you spend on microblogs?	0.496	−0.055	−0.510	0.674^**^
4. How often do you make new friends by microblogs?	0.049	0.317	−0.427	0.461^**^
9. How often do you feel the need to guard against or confidentialwhen someone asks what you do in microblogs?	0.299	0.033	−0.034	0.468^**^
10. How often do you feel microblogs can help you to comfort andresist the cares of life?	0.053	0.366	−0.080	0.500^**^
Percentage of variance explained	42.69%	7.35%	5.62%	

a Item numbers correspond to the original item number on the MEUS. **P<.01. The same as below.

The first factor accounted for 42.69% of the shared variance. 11 items had high loading that were associated with experiencing withdrawal effects and health problems due to excessive microblog use. The factor was hence termed “withdrawal and health problem.” Cronbach’s alpha for these 11 items was 0.930, indicating adequate internal consistency.

The second factor had 5 items, which were associated with interacting with others. This factor was termed “social comfort,” and it accounted for 7.35% of the shared variance. Cronbach’s alpha for these 5 items was 0.779, indicating good internal consistency.

The third factor had 7 items with high loadings, which were associated with negative effects on work or academic performance and mismanagement of time due to microblogging. This factor was termed “time management and performance,” and it accounted for 5.62% of the shared variance. Cronbach’s alpha for these 7 items was 0.864, indicating adequate internal consistency.

#### Reduction of numbers of items

Subsequently, we reviewed each item (statement or question) based on its weighting within the scale, its corrected item–total correlation value, and its meaning. We also considered the frequency of “no” answers for each item or question. 12 items (4 for each factor) were selected in order to reduce items and create a clear-cut factor structure for convenient use (see Table 3).

**Table 3:** Factor structure of the 12-item MEUS.

Factor 1 (withdrawal and health problem)	Factor 2 (social comfort)	Factor 3 (time management and performance)
Item 23	Item 25	Item 8
Item 21	Item 24	Item 7
Item 20	Item 27	Item 16
Item 19	Item 26	Item 6

### Psychometric Properties of the MEUS–Sample 2

#### EFA

The 12-item MEUS demonstrated good internal consistency (α = 0.835). Using the same criterion discussed above, we conducted an EFA in which we extracted three factors, which accounted for 63.18% of total variance. The specific item loadings on these three factors are listed in Table 4.

**Table 4:** Factor loadings from EFA of the 12-item MEUS.

Items	Factor 1 (withdrawal and health problem)	Factor 2 (social comfort)	Factor 3 (time management and performance)
21.	0.872	−0.071	0.026
23.	0.849	−0.112	0.045
6.	0.821	0.010	−0.017
19.	0.709	0.150	0.063
20.	0.525	0.330	0.077
25.	−0.271	0.822	0.032
27.	0.265	0.645	−0.024
26.	0.345	0.642	0.012
7.	−0.076	0.017	0.839
16.	0.013	0.219	0.797
8.	0.062	−0.173	0.761
24.	0.398	0.471	0.048

#### Reduction of numbers of items

Item 6 did not belong to the same factor in the two EFAs, and the loading of item 24 was lower than 0.5. As such we deleted these two items (see Table 5). Finally, we developed a 10-item questionnaire (see Supporting Information S3).

**Table 5:** Factor loadings from EFA of the 10-item MEUS.

Factor 1(withdrawal and health problem)	Factor 2(social comfort)	Factor 3(time management and performance)
Item 23	Item 25	Item 8
Item 21	Item 27	Item 7
Item 20	Item 26	Item 16
Item 19		

#### Correlations between subscales

The correlation of subscales with each other was above .211 (*p*<.001), and the correlation of the total scale with each of the subscales was greater than .678 (*p*<.001) (see Table 6).

**Table 6:** Correlations between subscales of MEUS.

	Factor 1(withdrawal and health problem)	Factor 2(social comfort)	Factor 3(time management and performance)
Factor 1	1	–	–
Factor 2	0.328**	1	–
Factor 3	0.473**	0.211**	1
The total score of MEUS	0.784**	0.704**	0.678**

^**^ *P*<.05.

### Psychometric Properties of the MEUS–Sample 3

#### Confirmatory factor analysis (CFA)–internal consistency of the MEUS

We found high reliabilities for each of the three subscales in Sample 3: “withdrawal and health problem” (α = .721), “social comfort” (α = .628), and “time management and performance” (α = .830). Cronbach’s alpha for the overall MEUS was .803.

#### Structure validity

**Figure 1:**
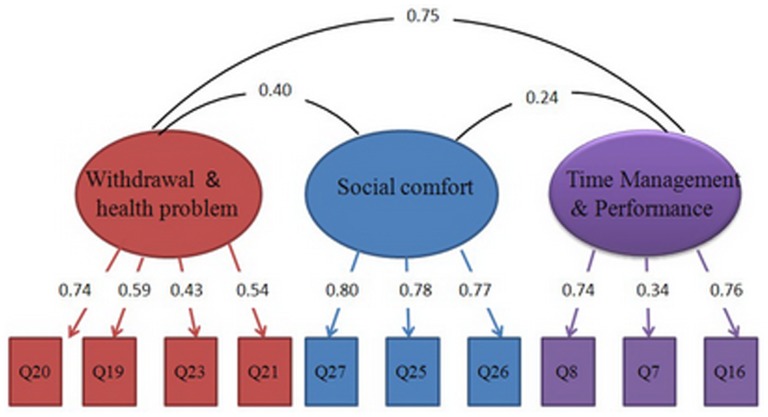
Factorial structure of the MEUS.

To verify the factor structure identified through EFA, CFA was performed on Sample 3. For the current CFA model, the ratio of chi-square to degrees of freedom was 5.5 (χ^2^ = 165.8; *df* = 30); the comparative fit index was .953; the normed fit index was .944; the root mean square error of approximation was .069; the relative fit index was .916; and the incremental fit index was .954, indicating adequate model fit. The model of factorial structure for the MEUS is shown in Fig. 1.

#### Criterion validity

To assess the criterion validity of these scales, the correlation between the total score from the MEUS and self-reporting of the degree of importance of and dependence on microblogs was calculated. It was significantly related to the self-report of the degree of the importance of (*ρ* = 0.504, *p*<.001) and dependence on (*ρ* = 0.624, *p*<.001) microblogs, suggesting that the scale has good criterion validity.

### Characteristics of College Students’ Excessive Use of Microblogs-Sample 3

#### Gender difference

We performed Mann-Whitney *U* tests to examine gender differences between total scores in Sample 3. Our results showed that female participants had significantly higher total scores compared to male participants (*U* = 92395.5, *p* = .002; male: *M* = 21.1, *SD* = 6.4; female: *M* = 22.4, *SD* = 6.2).

### Characteristics of Microblog Use and of Excessive Microblog Use-Sample 3

Sample 3 showed that the correlation between the total duration of microblogging (the total duration of microblogging means the period that a participant has been using Microblog up to the time of the study, e.g. how many months.), and the MEUS score is not significant (*ρ* = 0.077, *p* = .153).

**Figure 2:**
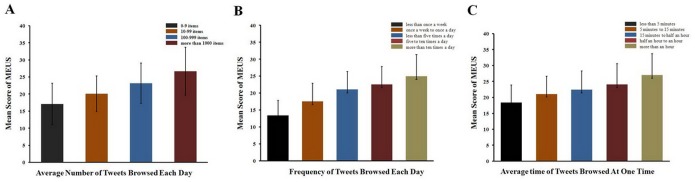
Relationship between the MEUS and the measurement of (a) average number of Tweets browsed, (b) frequency of browsing Twitter, and (c) average time browsing Twitter (per day).

We undertook analysis of variance to examine the difference between the average numbers of Tweets browsed per day in the total scores. Our results showed that there was a significant main effect among four levels of Tweets browsed (χ^2^ = 129.2, *p*<.001). Furthermore, the more Tweets a participant browsed, the higher the total scores (see Fig. 2a).

We applied the same analysis on the frequency of browsing Microblog per day and the average amount of time spent browsing Microblog on a single occasion. There was also significant main effect among five levels of browsing frequency (χ^2^ = 192.8, *p*<.001) and time levels (χ^2^ = 108.1, *p*<.001). The more frequently a participant browsed tweets a day, the higher the total score (see Fig. 2b), and the same applied to the average amount of time of spent browsing a microblog at any one time (see Fig. 2c).

In Sample 3, participants’ behavior in microblogs chiefly comprised posting new Tweets (84.1%), browsing the statuses and opinions of people they were following (91.7%), learning hot topics (77.2%), viewing Re-tweets, commenting or using the Twitter @ symbol (75.1%). By comparison, other behaviors, such as participating in discussion of hot topics (26.5%), proposing a topic (15.4%), making friends/expanding number of friends/communicating with famous people or senior personage (16.9%) represented less than 30%.

### Characteristics of Microblog Use and of Excessive Microblog Use-Sample 4

#### Excessive use of microblogs and Internet addiction

The total score from the MEUS significantly correlated with the score from Young’s IAT (*ρ = *0.581, *p*<.001). Compared to Young’s IAT, two factors (“withdrawal and health problem” and “time management and performance”) of the excessive use of microblogs are similar to those of Internet addiction. To confirm this, we computed the partial correlation between the excessive use of microblogs and Internet addiction while regressing out these two factors. Our results revealed that the original significant correlation between the score from the MEUS and that from the IAT vanished in this sample after controlling for these two factors (*r* = 0.087, *p* = .090).

### Characteristics of Microblog Use and of Excessive Microblog Use-Sample 5

#### Excessive use of microblogs, social interaction, and self-disclosure

Our results showed that the score from the MEUS had a significant positive correlation, with small effect (based on Cohen’s criterion [47]), with the score from the Real Social Interaction Scale (*ρ* = 0.106, *p* = .001), and with the score from the Self-disclosure Scale in reality (*ρ* = 0.189, *p*<.001). Further, the score of MEUS significantly positively correlated with the score of the Self-disclosure Scale on microblogs (*ρ* = 0.343, *p*<.001) with moderate effect [47].

### Norm of College Students’ Excessive Use of Microblogs

#### Norm sample distribution

We used Sample 3 and Sample 5 data to calculate the norm table including raw scores and standard scores of the MEUS (see Supporting Information S4). This norm comprised 1,949 participants; 1,110 males and 839 females (see Table 1). The subjects can find their positions in this norm table according to their scores on MEUS.

### Standard of the Excessive Use of Microblogs in College Students

**Figure 3:**
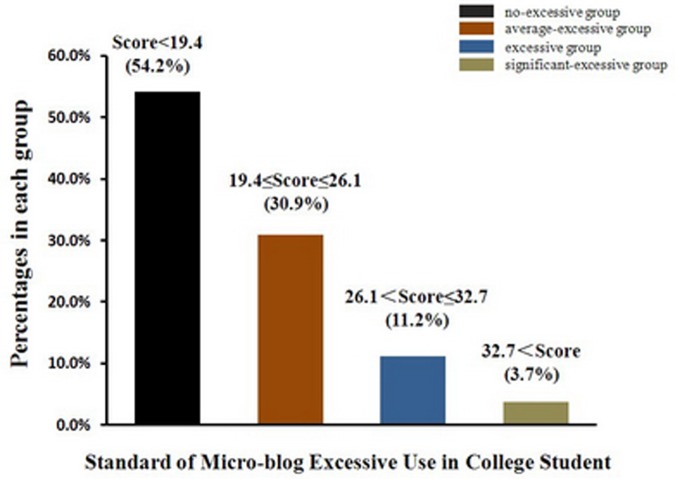
Standard of excessive use of microblogs among college students.

In the absence of a standard cutoff point, as the results could not be compared with either clinical research results or with other previously validated questionnaires, obtained score results were grouped according to their deviation from the mean (see Demetrovics et al., 2011) [48]. We created four groups from this norm. Participants with a score below the mean score (19.5) were assigned to the “non excessive use” (NEU) group. Those whose score was one standard deviation (6.6), at most, above the mean score were assigned to the “average excessive use” (AEU) group. Participants with a score that was more than one standard deviation above the mean score belonged either to the “excessive use” (EU) group (with a score less than two standard deviations above the mean) or to the “significant excessive use” (SEU) group (with a score more than two standard deviations above the mean). According to this standard, participants with addictive symptoms accounting for circa 4% fell into the SEU category (see Fig. 3).

Utilizing our proposed standards on the excessive use of microblogs and Young’s criteria for the IAT [31], we calculated the amount of overlap between microblog overuse and Internet addiction. We found that 11 participants in our study exhibited symptoms of both problematic use of microblogs and Internet addiction (see Supporting Information S5).

## Discussion

### Psychometric Properties of the MEUS

As previously noted, there is currently no scale available for assessing the excessive use of microblogs. In the present study, we developed a scale assessing microblog overuse in Chinese college students in four stages, encompassing interviews, preliminary tests, subject data collection, and reliability and validity data analysis. In addition, our psychometric examinations included an evaluation of the factor structure of the scale’s items (questions), its internal consistency, and its construct and criterion validity. Ultimately, we compiled a 10-item scale, composed of three factors termed “withdrawal and health problem,” “time management and performance,” and “social comfort.” Although some of the consistency coefficients of this 10-item scale were a little lower than .70, the total reliability of the scale reached .80. The EFA data found that all factor loadings in the project were over .50, and the cumulative rate of interpretation was more than 63%. Result of confirmatory factor analysis also met acceptable psychometric standards. Comparative fit index values of 0.90 and above conventionally represent good model fit. Overall, the MEUS showed adequate psychometric properties within the study’s sample of college students from China. With Twitter now the most popular microblog platform in the United States, in the future, this instrument should be assessed on Twitter using a larger and more representative sample. We envisage that relatively the same results would be obtained in Twitter.

### How Excessive is the “Excessive” Use of Microblogs?

We collected 1,949 nationwide norm data, which had strong representation of college students in China. Our data showed that 4%–15% of the participants were excessive users of microblogs, indicating that a certain proportion of college students may have symptoms of problematic microblog use, which relates to recent estimates that indicate that 4.07%–14.08% of college students in China have a predominantly inattentive subtype of Internet addiction disorder [49]–[53].

In this norm, participants with a score below 19.4 belonged to the NEU group. Those whose score was 19.4–26.1 belonged to the AEU group, which was characterized by mild symptoms of microblog overuse. Participants with a score 26.1–32.7 belonged to the EU group, whose members met some criterion of problematic microblog use with regard to withdrawal, time management. Those participants with a score of more than 32.7 (i.e., ≥33) fell into the SEU group, and are known as “microblog freaks” in China. They spend a lot of time on microblogs, and have a sense of dependency on microblogs exhibited in their behavior and psychology.

### Similarities between the Excessive Use of Microblogs and Internet addiction

Based on our data, we established that the excessive use of microblogs and Internet addiction are similar in two factors: “withdrawal and health problem” and “time management and performance problem.” These two factors are core components of Internet addiction or Facebook addiction, reflecting salience, loss of control, and withdrawal [16]. To the Internet addict, computer usage significantly preoccupies their time and thoughts [54]. As an Internet-based behavior, microblog usage is significantly predicted by an individual’s online behavior [55]. Thus, excessive users of microblogs show similar symptoms as Internet addicts. The “withdrawal effect” can be an indication of addictive properties, and the “time management problem” can be described as a control disorder like Internet addiction disorder. In fact, any form of excessive engagement in particular behavioral has some common characteristics, such as desire or impulse and loss of control [56], [57].

In addition, we did not find significant correlation between microblog use duration and excessive use of microblogs. This finding is similar to results in the field of Internet addiction; that is, there is no predictable association between total use time of internet and Internet addiction [58]. For the Internet addict, although computer usage significantly preoccupies their time and thoughts, normal computer use, such as working with a computer, can also take on an addictive quality at times [54]. Hilgard et al. (2013) suggest that a more effective way to prove whether an individual is an Internet game addict or not is to observe whether he or she always plays Internet games in their free time, rather than an analysis of the total usage time [58]. We assert that there is little relation between the total duration of microblogging and addiction, which means the duration of microblog use is not an indicator of microblog overuse [59].

The withdrawal effect, time management problems, and a lack of correlation between usage time and Internet addiction are an indication of addictive tendencies [21]. This further suggests that both Internet addiction and excessive use of microblogs have similar psychological mechanisms; in this case, the relevant element of addiction.

Furthermore, since 11 participants (out of 15 IA participants and 74 MEU participants) in our study exhibited symptoms of both problematic use of microblogs and Internet addiction, we may conclude that the excessive use of microblogs has some significant similarities with Internet addiction.

### Differences between the Excessive Use of Microblogs and Internet addiction

In the present study, we identified three differences between the excessive use of microblogs and Internet addiction. First, comparing the factors of the MEUS and Young’s IAT, we found the “social comfort” factor to be unique in the MEUS (in the IAT, it is the “reality substitute” factor). After controlling for the “withdrawal and health problem” and “time management and performance” factors, the significant correlation between MEUS and IAT disappeared. These findings indicate that “social comfort” may be a vital factor in measuring the excessive use of microblogs. The motivation for people using social networks, be it Twitter or Facebook, is to seek social comfort and reduce loneliness [60], while individuals play Internet games more for realistic alternative [30]. Second, we found that females scored significantly higher in the MEUS than males. This is consistent with Smith and Rainie’s (2010) research showing that women are more likely to use Twitter than men [61]. However, this is contrary to the finding of Internet addiction. Numerous research results on Internet addiction show that males get higher scores than females [62]–[66]. Compared to traditional Internet surfing, writing and posting tweets by young adults is more about self-presentation [38]. In addition, several studies have shown that females do tend to disclose more information than males do [67]–[69]. Further, in a meta-analysis of 205 studies, Dindia and Allen examined gender differences in self-disclosure and found that females disclose more personal information than males [70]. In our study, the results showed significant positive association between the excessive use of microblogs and self-disclosure. This is consistent with other results (see [38], [39]) that show that self-presentation is a major drive for actively participating in social networking sites [39]. The difference in self-presentation between males and females suggests the essential role of social comfort, hence its importance in our MEUS.

The last difference between Internet addiction and the excessive use of microblogs concerns social interactions. There are different forms of motivation that could explain Internet-based behaviors, such as interpersonal interaction, social support, and self-fulfillment [71]–[73]. Internet addiction leads to significant reductions of social interaction in reality among college students [74], as well as to the impairment of real-life social relationships [75]–[78]. Individuals may spend a lot of time surfing the Internet in order to avoid relationships in real life. This indicates a lack of skills and energy to build relationships in real life [79]. However, our study reveals that excessive use of microblogs has positive correlation with real-life social interactions. This suggests that microblogs may contribute to or not hinder the development of personal relations in real life [5]. Further, the real-time personal updates featured in Twitter and other microblogs may help to sustain a virtual feeling of proximity facilitating social connectedness (i.e., being there, still there) [80]. Thus, as recent research has identified, “the line between the physical world and online social realities has been blurred by the new possibilities afforded by real-time communication and broadcasting, which appear to greatly enhance our social and cognitive capabilities in establishing and maintaining social relations” [81]. Arguably, this is further augmented by a combination of mobile communication devices with new tools such as Twitter [82]. Hence, individuals can obtain social comfort from improving relationships-a notable difference between microblog overuse and Internet addiction.

In summary, from the perspective of factor analysis, gender differences, self-disclosure, and interpersonal interactions, “social comfort” is the main distinguishing factor of the excessive use of microblogs from Internet addiction. This also indicates the difference between microblog overuse and Internet addiction.

On one hand, Internet addiction is not regarded as homogeneous and several subtypes have been identified, such as excessive gaming, online sexual pre-occupation, and e-mailing/texting [83]. On the other hand, some researchers have argued that excessive use of different types of Internet applications reveal a single form of Internet addiction [15]. At the same time, with the development of technology, increasing numbers of popular network applications, including microblogs, are being invented. MySpace and Facebook, representing online social networking, have gone through a period of high-speed growth, while the rapid development of microblogging, with its social function, has become a key factor to its replacement of the social networking sites [3]. Furthermore, even newer communication platforms and media such as micro-letters like Whatapp are becoming more and more popular in China today [84]. Results of the present study indicate that excessive use of microblogs and Internet addiction are not exactly the same, suggesting that some symptoms of Internet addiction may change due to development of new technologies. Moreover, Internet addiction may be different from other forms of addiction, such as substance abuse and impulse control disorder. It is not a “fixed” disease awaiting our exploration, but rather a changing and evolving disorder.

This alteration of the concept of Internet addiction may also reflect changes in the Internet itself. For the past two to three decades, people have generally thought that Internet use could damage social relations in real life. Weiser’s (2001) longitudinal study of college students showed a significant negative effect of spending more hours using the Internet, in terms of personal loneliness, depression, and life satisfaction [85]. Researchers thought that online communication reduced real human engagement [86], communication time with family and friends [87], and real social circles [88]. For teenagers, it was thought that their relationships with family and friends could become weaker through increased levels of Internet communication [89]. Online relationships rarely developed into real social relations networks, and so, compared to the latter, online relationships were considered to be very superficial and unsustainable [90], [91]. Conversely, recent research, especially that following the rise of social network sites (Facebook in 2004 and Twitter in 2006), suggests just the opposite. For example, youngsters who actively participate in online creation and sharing are reportedly more involved in additional extracurricular activities in their real life [92]. Koutamanis and colleagues reported that instant messaging can increase teenagers’ capacity for friendship initiation [93]; young adults can practice their social skills online, and, with the passage of time, may have better friendships in real life. Our results suggest the same thing, with the change from “reality substitute” to “social comfort.” Both “reality substitute” and “social comfort” reflect the influence of internet use on real social interaction–the distinction to be drawn is whether this is a positive or a negative influence. Two further studies have shown that the size of an individual’s online social network is related to the structure of specific, socialization-related areas of their brain (e.g., the amygdala, the left middle temporal gyrus, the right posterior superior temporal sulcus, and the right entorhinal cortex) [94], [95]. Our results and those from previously cited studies suggest that Internet use is being incorporated into real life more and more, developing from being considered impairment to our social skills to being a tool for our social skills. Moreover, our study suggests that the effect of the Internet may continue to change and become more complex, and so requires further attention and study.

## Limitations and Conclusion

The study had some limitations that merit consideration. Firstly, data for the study were collected through an online survey and geographically, participants were mainly from universities in Eastern China. The sample may not be a correct representation of colleges in China. However, most of the universities at which the study was undertaken are national colleges that attract students from all regions of China. As such, we are confident that the sample correctly represented the microblogging tendencies of college students in China.

Secondly, the study exclusively used self-report questionnaires in data collection. Self-reports are sometimes biased and not entirely reliable. Participants may have understated the extent of their internet and microblog use; presenting less severe symptoms of withdrawal, tolerance and/or social problems. As a result, our findings may not provide a complete picture of the extent of excessive microblogging in China. Future studies should incorporate information provided by peers, guardians and teachers as well as clinical data and employ cognitive behavior tasks.

Thirdly, at present, we just collected data of normal population, and the results showed that 11 participants in our study exhibited symptoms of both problematic use of microblogs and Internet addiction. So, we are planning to collect clinical samples to further verify the validity of the scale, and also to provide a reference threshold for clinical evaluation. And then, we will work on the neural basis of the microblog excessive use to provide a complete picture of the extent of excessive microblogging.

In summary, the three-factor MEUS developed in the current study appeared to be adequate with regard to this sample, which showed good internal consistency as well as construct and criterion validity. Although there are some similarities between excessive use of microblogs and Internet addiction, the MEUS has a unique factor–“social comfort”–, compared to Young’s IAT [28]. Further, the MEUS had significant positive correlation with self-disclosure and social interaction, which is the reverse of published findings on Internet addiction. Our study suggests that excessive use of microblogs is not exactly the same as Internet addiction; hence, it would be illogical to measure the two using the same scale. Finally, the pathogenesis and potential treatment of problematic use of microblogs and Internet addiction may not be fully employed in the same way, an area in which warrants further research in future.

## Supporting Information

Supporting Information S1 Demographic Information.
(DOCX)

Supporting Information S2 Interview Questions.
(DOCX)

Supporting Information S3 Microblog Excessive Use Scale.
(DOCX)

Supporting Information S4 Norm Table.
(DOCX)

Supporting Information S5 The overlap of Micro-blog excessive use (MEU) and Internet addiction (IA).
(DOCX)
