# Supplementary material for: Is the Excessive Use of Microblogs an Internet Addiction? Developing a Scale for Assessing the Excessive Use of Microblogs in Chinese College Students
Source: PLoS One. 2014 Nov 18;9(11):e110960. doi: 10.1371/journal.pone.0110960 (PMC4236055; doi:10.1371/journal.pone.0110960)
Supplement: Supporting Information S1 — Demographic Information. (DOCX) [file pone.0110960.s001.docx]

Is the Excessive Use of Microblogs an Internet Addiction? Developing a Scale for Assessing the Excessive Use of Microblogs in Chinese College Students

Juan Hou^a1^, Zhichao Huang^a2^, Hongxia Li^a3^, Mengqiu Liu^4^, Wei Zhang^2^, Ning Ma^2^, Lizhuang Yang^2^, Feng Gu^2^, Ying Liu^4^, Shenghua Jin^3^, Xiaochu Zhang*^2,5^

**Demographic Information**

1. Age___

2. Sex___

3. Education background

a. undergraduate b. graduate

4. The first time you use Micro‐blog:_______(mm/dd/yyyy)

5. So far, the number of followers＿, tweets＿, followings＿

6. The average number of tweets you browse a day?

a. 0‐9 b. 10‐99 c. 100‐999 d. 1000 or above

7. The frequency you browse tweets a day?

a. Less than once a week

b. Once a week to once a day

c. Less than five times a day

d. Five to ten times a day

e. More than ten times a day

8. The average time you browse Micro‐blog one time?

a. less than five minutes

b. five minutes to 15minutes

c.15 minutes to half an hour

d. half an hour to an hour

e. more than an hour

9. Do you view all daily updates of your followings?

a. yes b. no

10. Would you spend money to increase your follower count?

a. yes b. no

11. Your behavior in the Micro‐blog (multiple‐choice)

a. compose new tweet

b. browse followings’ status and opinions

c. learn hot topics

d. participate in discussing hot topics

e. view the retweet, commentor@

f. propose a topic

g. make friends, expand society of friends, communicate with famous person, senior personage

h. others

12. Use 1 to 10 to score the importance of Micro‐blog for you.______

1 means “not important”, 10 means “very important”

13. Use 1 to 10 to express the degree you depend on Micro‐blog______

1 means “not important”,10 means “very important”
