# Supplementary material for: Is the Excessive Use of Microblogs an Internet Addiction? Developing a Scale for Assessing the Excessive Use of Microblogs in Chinese College Students
Source: PLoS One. 2014 Nov 18;9(11):e110960. doi: 10.1371/journal.pone.0110960 (PMC4236055; doi:10.1371/journal.pone.0110960)
Supplement: Supporting Information S2 — Interview Questions. (DOCX) [file pone.0110960.s002.docx]

Is the Excessive Use of Microblogs an Internet Addiction? Developing a Scale for Assessing the Excessive Use of Microblogs in Chinese College Students

Juan Hou^a1^, Zhichao Huang^a2^, Hongxia Li^a3^, Mengqiu Liu^4^, Wei Zhang^2^, Ning Ma^2^, Lizhuang Yang^2^, Feng Gu^2^, Ying Liu^4^, Shenghua Jin^3^, Xiaochu Zhang*^2,5^

**Interview Questions**

1. Your behavior in the Micro‐blog. Why these activities attract you?

2. Talk about your feelings of using Micro‐blog.

3. Talk about your relationship with others.

4. Under what circumstances would you like use the Micro‐blog?
