# Supplementary material for: Is the Excessive Use of Microblogs an Internet Addiction? Developing a Scale for Assessing the Excessive Use of Microblogs in Chinese College Students
Source: PLoS One. 2014 Nov 18;9(11):e110960. doi: 10.1371/journal.pone.0110960 (PMC4236055; doi:10.1371/journal.pone.0110960)
Supplement: Supporting Information S3 — Microblog Excessive Use Scale. (DOCX) [file pone.0110960.s003.docx]

Is the Excessive Use of Microblogs an Internet Addiction? Developing a Scale for Assessing the Excessive Use of Microblogs in Chinese College Students

Juan Hou^a1^, Zhichao Huang^a2^, Hongxia Li^a3^, Mengqiu Liu^4^, Wei Zhang^2^, Ning Ma^2^, Lizhuang Yang^2^, Feng Gu^2^, Ying Liu^4^, Shenghua Jin^3^, Xiaochu Zhang*^2,5^

**Microblog Excessive Use Scale**

Below is the condition of twitter use,please reply every item according to yourself. “1”means never,“2”means seldom,“3”means sometimes,“4”means often, “5”means usually,“6”means always .

|  | never | seldom | sometimes | often | usually | always |
| --- | --- | --- | --- | --- | --- | --- |
| How often do you find yourself saying “just a few more minutes” when using microblogs? | 1 | 2 | 3 | 4 | 5 | 6 |
| How often would you try to increase your followers unconsciously by all means? | 1 | 2 | 3 | 4 | 5 | 6 |
| How often do you feel depressed, moody, or nervous when you are off microblogs? | 1 | 2 | 3 | 4 | 5 | 6 |
| How is your health affected by using microblogs? | 1 | 2 | 3 | 4 | 5 | 6 |
| How often do you check your microblog before something else that you need to do? | 1 | 2 | 3 | 4 | 5 | 6 |
| How often do you sleep less than four hours a day because of using microblogs? | 1 | 2 | 3 | 4 | 5 | 6 |
| How often do you feel excited when others Retweet your Tweets or comment on your microblogs? | 1 | 2 | 3 | 4 | 5 | 6 |
| How often does your job performance or productivity suffer because of your use of microblogs? | 1 | 2 | 3 | 4 | 5 | 6 |
| How often do you choose to spend more time on microblogs over going out with others? | 1 | 2 | 3 | 4 | 5 | 6 |
| How often do you get more followers by increasing the number that you are following? | 1 | 2 | 3 | 4 | 5 | 6 |
