# Supplementary material for: Is the Excessive Use of Microblogs an Internet Addiction? Developing a Scale for Assessing the Excessive Use of Microblogs in Chinese College Students
Source: PLoS One. 2014 Nov 18;9(11):e110960. doi: 10.1371/journal.pone.0110960 (PMC4236055; doi:10.1371/journal.pone.0110960)
Supplement: Supporting Information S4 — Norm Table. (DOCX) [file pone.0110960.s004.docx]

Is the Excessive Use of Microblogs an Internet Addiction? Developing a Scale for Assessing the Excessive Use of Microblogs in Chinese College Students

Juan Hou^a1^, Zhichao Huang^a2^, Hongxia Li^a3^, Mengqiu Liu^4^, Wei Zhang^2^, Ning Ma^2^, Lizhuang Yang^2^, Feng Gu^2^, Ying Liu^4^, Shenghua Jin^3^, Xiaochu Zhang*^2,5^

**Norm Table**

| **Score** | **Z** | **T** | **Score** | **Z** | **T** | **Score** | **Z** | **T** |
| --- | --- | --- | --- | --- | --- | --- | --- | --- |
| 56.00 | 3.49 | 84.87 | 22.00 | 0.58 | 55.76 | 16.00 | -0.24 | 47.62 |
| 47.00 | 3.18 | 81.81 | 22.00 | 0.58 | 55.76 | 16.00 | -0.24 | 47.62 |
| 45.00 | 3.03 | 80.30 | 22.00 | 0.58 | 55.76 | 16.00 | -0.24 | 47.62 |
| 44.00 | 2.93 | 79.27 | 22.00 | 0.58 | 55.76 | 16.00 | -0.24 | 47.62 |
| 44.00 | 2.93 | 79.27 | 22.00 | 0.58 | 55.76 | 16.00 | -0.24 | 47.62 |
| 44.00 | 2.93 | 79.27 | 22.00 | 0.58 | 55.76 | 16.00 | -0.24 | 47.62 |
| 43.00 | 2.73 | 77.29 | 22.00 | 0.58 | 55.76 | 16.00 | -0.24 | 47.62 |
| 43.00 | 2.73 | 77.29 | 22.00 | 0.58 | 55.76 | 16.00 | -0.24 | 47.62 |
| 42.00 | 2.64 | 76.39 | 22.00 | 0.58 | 55.76 | 16.00 | -0.24 | 47.62 |
| 41.00 | 2.60 | 76.01 | 22.00 | 0.58 | 55.76 | 16.00 | -0.24 | 47.62 |
| 41.00 | 2.60 | 76.01 | 22.00 | 0.58 | 55.76 | 16.00 | -0.24 | 47.62 |
| 40.00 | 2.53 | 75.35 | 22.00 | 0.58 | 55.76 | 16.00 | -0.24 | 47.62 |
| 40.00 | 2.53 | 75.35 | 22.00 | 0.58 | 55.76 | 16.00 | -0.24 | 47.62 |
| 39.00 | 2.48 | 74.78 | 22.00 | 0.58 | 55.76 | 16.00 | -0.24 | 47.62 |
| 39.00 | 2.48 | 74.78 | 22.00 | 0.58 | 55.76 | 16.00 | -0.24 | 47.62 |
| 39.00 | 2.48 | 74.78 | 22.00 | 0.58 | 55.76 | 16.00 | -0.24 | 47.62 |
| 38.00 | 2.41 | 74.06 | 22.00 | 0.58 | 55.76 | 16.00 | -0.24 | 47.62 |
| 38.00 | 2.41 | 74.06 | 22.00 | 0.58 | 55.76 | 16.00 | -0.24 | 47.62 |
| 38.00 | 2.41 | 74.06 | 22.00 | 0.58 | 55.76 | 16.00 | -0.24 | 47.62 |
| 38.00 | 2.41 | 74.06 | 22.00 | 0.58 | 55.76 | 16.00 | -0.24 | 47.62 |
| 38.00 | 2.41 | 74.06 | 22.00 | 0.58 | 55.76 | 16.00 | -0.24 | 47.62 |
| 37.00 | 2.31 | 73.08 | 22.00 | 0.58 | 55.76 | 16.00 | -0.24 | 47.62 |
| 37.00 | 2.31 | 73.08 | 22.00 | 0.58 | 55.76 | 16.00 | -0.24 | 47.62 |
| 37.00 | 2.31 | 73.08 | 22.00 | 0.58 | 55.76 | 15.00 | -0.38 | 46.22 |
| 37.00 | 2.31 | 73.08 | 22.00 | 0.58 | 55.76 | 15.00 | -0.38 | 46.22 |
| 37.00 | 2.31 | 73.08 | 22.00 | 0.58 | 55.76 | 15.00 | -0.38 | 46.22 |
| 37.00 | 2.31 | 73.08 | 22.00 | 0.58 | 55.76 | 15.00 | -0.38 | 46.22 |
| 37.00 | 2.31 | 73.08 | 22.00 | 0.58 | 55.76 | 15.00 | -0.38 | 46.22 |
| 36.00 | 2.20 | 71.99 | 22.00 | 0.58 | 55.76 | 15.00 | -0.38 | 46.22 |
| 36.00 | 2.20 | 71.99 | 22.00 | 0.58 | 55.76 | 15.00 | -0.38 | 46.22 |
| 36.00 | 2.20 | 71.99 | 22.00 | 0.58 | 55.76 | 15.00 | -0.38 | 46.22 |
| 35.00 | 2.16 | 71.60 | 21.00 | 0.43 | 54.31 | 15.00 | -0.38 | 46.22 |
| 35.00 | 2.16 | 71.60 | 21.00 | 0.43 | 54.31 | 15.00 | -0.38 | 46.22 |
| 35.00 | 2.16 | 71.60 | 21.00 | 0.43 | 54.31 | 15.00 | -0.38 | 46.22 |
| 35.00 | 2.16 | 71.60 | 21.00 | 0.43 | 54.31 | 15.00 | -0.38 | 46.22 |
| 35.00 | 2.16 | 71.60 | 21.00 | 0.43 | 54.31 | 15.00 | -0.38 | 46.22 |
| 35.00 | 2.16 | 71.60 | 21.00 | 0.43 | 54.31 | 15.00 | -0.38 | 46.22 |
| 35.00 | 2.16 | 71.60 | 21.00 | 0.43 | 54.31 | 15.00 | -0.38 | 46.22 |
| 35.00 | 2.16 | 71.60 | 21.00 | 0.43 | 54.31 | 15.00 | -0.38 | 46.22 |
| 35.00 | 2.16 | 71.60 | 21.00 | 0.43 | 54.31 | 15.00 | -0.38 | 46.22 |
| 35.00 | 2.16 | 71.60 | 21.00 | 0.43 | 54.31 | 15.00 | -0.38 | 46.22 |
| 35.00 | 2.16 | 71.60 | 21.00 | 0.43 | 54.31 | 15.00 | -0.38 | 46.22 |
| 35.00 | 2.16 | 71.60 | 21.00 | 0.43 | 54.31 | 15.00 | -0.38 | 46.22 |
| 35.00 | 2.16 | 71.60 | 21.00 | 0.43 | 54.31 | 15.00 | -0.38 | 46.22 |
| 35.00 | 2.16 | 71.60 | 21.00 | 0.43 | 54.31 | 15.00 | -0.38 | 46.22 |
| 35.00 | 2.16 | 71.60 | 21.00 | 0.43 | 54.31 | 15.00 | -0.38 | 46.22 |
| 35.00 | 2.16 | 71.60 | 21.00 | 0.43 | 54.31 | 15.00 | -0.38 | 46.22 |
| 35.00 | 2.16 | 71.60 | 21.00 | 0.43 | 54.31 | 15.00 | -0.38 | 46.22 |
| 35.00 | 2.16 | 71.60 | 21.00 | 0.43 | 54.31 | 15.00 | -0.38 | 46.22 |
| 35.00 | 2.16 | 71.60 | 21.00 | 0.43 | 54.31 | 15.00 | -0.38 | 46.22 |
| 34.00 | 1.97 | 69.65 | 21.00 | 0.43 | 54.31 | 15.00 | -0.38 | 46.22 |
| 34.00 | 1.97 | 69.65 | 21.00 | 0.43 | 54.31 | 15.00 | -0.38 | 46.22 |
| 34.00 | 1.97 | 69.65 | 21.00 | 0.43 | 54.31 | 15.00 | -0.38 | 46.22 |
| 34.00 | 1.97 | 69.65 | 21.00 | 0.43 | 54.31 | 15.00 | -0.38 | 46.22 |
| 34.00 | 1.97 | 69.65 | 21.00 | 0.43 | 54.31 | 15.00 | -0.38 | 46.22 |
| 34.00 | 1.97 | 69.65 | 21.00 | 0.43 | 54.31 | 15.00 | -0.38 | 46.22 |
| 34.00 | 1.97 | 69.65 | 21.00 | 0.43 | 54.31 | 15.00 | -0.38 | 46.22 |
| 34.00 | 1.97 | 69.65 | 21.00 | 0.43 | 54.31 | 15.00 | -0.38 | 46.22 |
| 34.00 | 1.97 | 69.65 | 21.00 | 0.43 | 54.31 | 15.00 | -0.38 | 46.22 |
| 34.00 | 1.97 | 69.65 | 21.00 | 0.43 | 54.31 | 15.00 | -0.38 | 46.22 |
| 34.00 | 1.97 | 69.65 | 21.00 | 0.43 | 54.31 | 15.00 | -0.38 | 46.22 |
| 33.00 | 1.88 | 68.80 | 21.00 | 0.43 | 54.31 | 15.00 | -0.38 | 46.22 |
| 33.00 | 1.88 | 68.80 | 21.00 | 0.43 | 54.31 | 15.00 | -0.38 | 46.22 |
| 33.00 | 1.88 | 68.80 | 21.00 | 0.43 | 54.31 | 15.00 | -0.38 | 46.22 |
| 33.00 | 1.88 | 68.80 | 21.00 | 0.43 | 54.31 | 15.00 | -0.38 | 46.22 |
| 33.00 | 1.88 | 68.80 | 21.00 | 0.43 | 54.31 | 15.00 | -0.38 | 46.22 |
| 33.00 | 1.88 | 68.80 | 21.00 | 0.43 | 54.31 | 15.00 | -0.38 | 46.22 |
| 33.00 | 1.88 | 68.80 | 21.00 | 0.43 | 54.31 | 15.00 | -0.38 | 46.22 |
| 33.00 | 1.88 | 68.80 | 21.00 | 0.43 | 54.31 | 15.00 | -0.38 | 46.22 |
| 33.00 | 1.88 | 68.80 | 21.00 | 0.43 | 54.31 | 15.00 | -0.38 | 46.22 |
| 33.00 | 1.88 | 68.80 | 21.00 | 0.43 | 54.31 | 15.00 | -0.38 | 46.22 |
| 32.00 | 1.81 | 68.12 | 21.00 | 0.43 | 54.31 | 15.00 | -0.38 | 46.22 |
| 32.00 | 1.81 | 68.12 | 21.00 | 0.43 | 54.31 | 15.00 | -0.38 | 46.22 |
| 32.00 | 1.81 | 68.12 | 21.00 | 0.43 | 54.31 | 15.00 | -0.38 | 46.22 |
| 32.00 | 1.81 | 68.12 | 21.00 | 0.43 | 54.31 | 15.00 | -0.38 | 46.22 |
| 32.00 | 1.81 | 68.12 | 21.00 | 0.43 | 54.31 | 15.00 | -0.38 | 46.22 |
| 32.00 | 1.81 | 68.12 | 21.00 | 0.43 | 54.31 | 15.00 | -0.38 | 46.22 |
| 32.00 | 1.81 | 68.12 | 21.00 | 0.43 | 54.31 | 15.00 | -0.38 | 46.22 |
| 32.00 | 1.81 | 68.12 | 21.00 | 0.43 | 54.31 | 15.00 | -0.38 | 46.22 |
| 32.00 | 1.81 | 68.12 | 21.00 | 0.43 | 54.31 | 15.00 | -0.38 | 46.22 |
| 32.00 | 1.81 | 68.12 | 21.00 | 0.43 | 54.31 | 15.00 | -0.38 | 46.22 |
| 32.00 | 1.81 | 68.12 | 21.00 | 0.43 | 54.31 | 15.00 | -0.38 | 46.22 |
| 32.00 | 1.81 | 68.12 | 21.00 | 0.43 | 54.31 | 15.00 | -0.38 | 46.22 |
| 32.00 | 1.81 | 68.12 | 21.00 | 0.43 | 54.31 | 15.00 | -0.38 | 46.22 |
| 32.00 | 1.81 | 68.12 | 21.00 | 0.43 | 54.31 | 15.00 | -0.38 | 46.22 |
| 32.00 | 1.81 | 68.12 | 21.00 | 0.43 | 54.31 | 15.00 | -0.38 | 46.22 |
| 32.00 | 1.81 | 68.12 | 21.00 | 0.43 | 54.31 | 15.00 | -0.38 | 46.22 |
| 32.00 | 1.81 | 68.12 | 21.00 | 0.43 | 54.31 | 15.00 | -0.38 | 46.22 |
| 32.00 | 1.81 | 68.12 | 21.00 | 0.43 | 54.31 | 15.00 | -0.38 | 46.22 |
| 32.00 | 1.81 | 68.12 | 21.00 | 0.43 | 54.31 | 15.00 | -0.38 | 46.22 |
| 32.00 | 1.81 | 68.12 | 21.00 | 0.43 | 54.31 | 15.00 | -0.38 | 46.22 |
| 32.00 | 1.81 | 68.12 | 21.00 | 0.43 | 54.31 | 15.00 | -0.38 | 46.22 |
| 32.00 | 1.81 | 68.12 | 21.00 | 0.43 | 54.31 | 15.00 | -0.38 | 46.22 |
| 32.00 | 1.81 | 68.12 | 21.00 | 0.43 | 54.31 | 15.00 | -0.38 | 46.22 |
| 32.00 | 1.81 | 68.12 | 21.00 | 0.43 | 54.31 | 15.00 | -0.38 | 46.22 |
| 32.00 | 1.81 | 68.12 | 21.00 | 0.43 | 54.31 | 15.00 | -0.38 | 46.22 |
| 31.00 | 1.67 | 66.73 | 21.00 | 0.43 | 54.31 | 15.00 | -0.38 | 46.22 |
| 31.00 | 1.67 | 66.73 | 21.00 | 0.43 | 54.31 | 15.00 | -0.38 | 46.22 |
| 31.00 | 1.67 | 66.73 | 21.00 | 0.43 | 54.31 | 15.00 | -0.38 | 46.22 |
| 31.00 | 1.67 | 66.73 | 21.00 | 0.43 | 54.31 | 15.00 | -0.38 | 46.22 |
| 31.00 | 1.67 | 66.73 | 21.00 | 0.43 | 54.31 | 15.00 | -0.38 | 46.22 |
| 31.00 | 1.67 | 66.73 | 21.00 | 0.43 | 54.31 | 15.00 | -0.38 | 46.22 |
| 31.00 | 1.67 | 66.73 | 21.00 | 0.43 | 54.31 | 15.00 | -0.38 | 46.22 |
| 31.00 | 1.67 | 66.73 | 21.00 | 0.43 | 54.31 | 15.00 | -0.38 | 46.22 |
| 31.00 | 1.67 | 66.73 | 21.00 | 0.43 | 54.31 | 15.00 | -0.38 | 46.22 |
| 31.00 | 1.67 | 66.73 | 21.00 | 0.43 | 54.31 | 15.00 | -0.38 | 46.22 |
| 31.00 | 1.67 | 66.73 | 21.00 | 0.43 | 54.31 | 15.00 | -0.38 | 46.22 |
| 31.00 | 1.67 | 66.73 | 21.00 | 0.43 | 54.31 | 15.00 | -0.38 | 46.22 |
| 31.00 | 1.67 | 66.73 | 21.00 | 0.43 | 54.31 | 15.00 | -0.38 | 46.22 |
| 31.00 | 1.67 | 66.73 | 21.00 | 0.43 | 54.31 | 15.00 | -0.38 | 46.22 |
| 31.00 | 1.67 | 66.73 | 21.00 | 0.43 | 54.31 | 15.00 | -0.38 | 46.22 |
| 31.00 | 1.67 | 66.73 | 21.00 | 0.43 | 54.31 | 15.00 | -0.38 | 46.22 |
| 31.00 | 1.67 | 66.73 | 21.00 | 0.43 | 54.31 | 15.00 | -0.38 | 46.22 |
| 31.00 | 1.67 | 66.73 | 21.00 | 0.43 | 54.31 | 14.00 | -0.50 | 45.01 |
| 31.00 | 1.67 | 66.73 | 21.00 | 0.43 | 54.31 | 14.00 | -0.50 | 45.01 |
| 31.00 | 1.67 | 66.73 | 21.00 | 0.43 | 54.31 | 14.00 | -0.50 | 45.01 |
| 31.00 | 1.67 | 66.73 | 21.00 | 0.43 | 54.31 | 14.00 | -0.50 | 45.01 |
| 31.00 | 1.67 | 66.73 | 21.00 | 0.43 | 54.31 | 14.00 | -0.50 | 45.01 |
| 31.00 | 1.67 | 66.73 | 21.00 | 0.43 | 54.31 | 14.00 | -0.50 | 45.01 |
| 30.00 | 1.57 | 65.68 | 21.00 | 0.43 | 54.31 | 14.00 | -0.50 | 45.01 |
| 30.00 | 1.57 | 65.68 | 21.00 | 0.43 | 54.31 | 14.00 | -0.50 | 45.01 |
| 30.00 | 1.57 | 65.68 | 21.00 | 0.43 | 54.31 | 14.00 | -0.50 | 45.01 |
| 30.00 | 1.57 | 65.68 | 21.00 | 0.43 | 54.31 | 14.00 | -0.50 | 45.01 |
| 30.00 | 1.57 | 65.68 | 21.00 | 0.43 | 54.31 | 14.00 | -0.50 | 45.01 |
| 30.00 | 1.57 | 65.68 | 21.00 | 0.43 | 54.31 | 14.00 | -0.50 | 45.01 |
| 30.00 | 1.57 | 65.68 | 21.00 | 0.43 | 54.31 | 14.00 | -0.50 | 45.01 |
| 30.00 | 1.57 | 65.68 | 21.00 | 0.43 | 54.31 | 14.00 | -0.50 | 45.01 |
| 30.00 | 1.57 | 65.68 | 21.00 | 0.43 | 54.31 | 14.00 | -0.50 | 45.01 |
| 30.00 | 1.57 | 65.68 | 21.00 | 0.43 | 54.31 | 14.00 | -0.50 | 45.01 |
| 30.00 | 1.57 | 65.68 | 20.00 | 0.30 | 53.02 | 14.00 | -0.50 | 45.01 |
| 30.00 | 1.57 | 65.68 | 20.00 | 0.30 | 53.02 | 14.00 | -0.50 | 45.01 |
| 30.00 | 1.57 | 65.68 | 20.00 | 0.30 | 53.02 | 14.00 | -0.50 | 45.01 |
| 30.00 | 1.57 | 65.68 | 20.00 | 0.30 | 53.02 | 14.00 | -0.50 | 45.01 |
| 30.00 | 1.57 | 65.68 | 20.00 | 0.30 | 53.02 | 14.00 | -0.50 | 45.01 |
| 30.00 | 1.57 | 65.68 | 20.00 | 0.30 | 53.02 | 14.00 | -0.50 | 45.01 |
| 30.00 | 1.57 | 65.68 | 20.00 | 0.30 | 53.02 | 14.00 | -0.50 | 45.01 |
| 30.00 | 1.57 | 65.68 | 20.00 | 0.30 | 53.02 | 14.00 | -0.50 | 45.01 |
| 30.00 | 1.57 | 65.68 | 20.00 | 0.30 | 53.02 | 14.00 | -0.50 | 45.01 |
| 30.00 | 1.57 | 65.68 | 20.00 | 0.30 | 53.02 | 14.00 | -0.50 | 45.01 |
| 30.00 | 1.57 | 65.68 | 20.00 | 0.30 | 53.02 | 14.00 | -0.50 | 45.01 |
| 30.00 | 1.57 | 65.68 | 20.00 | 0.30 | 53.02 | 14.00 | -0.50 | 45.01 |
| 30.00 | 1.57 | 65.68 | 20.00 | 0.30 | 53.02 | 14.00 | -0.50 | 45.01 |
| 30.00 | 1.57 | 65.68 | 20.00 | 0.30 | 53.02 | 14.00 | -0.50 | 45.01 |
| 30.00 | 1.57 | 65.68 | 20.00 | 0.30 | 53.02 | 14.00 | -0.50 | 45.01 |
| 30.00 | 1.57 | 65.68 | 20.00 | 0.30 | 53.02 | 14.00 | -0.50 | 45.01 |
| 30.00 | 1.57 | 65.68 | 20.00 | 0.30 | 53.02 | 14.00 | -0.50 | 45.01 |
| 30.00 | 1.57 | 65.68 | 20.00 | 0.30 | 53.02 | 14.00 | -0.50 | 45.01 |
| 30.00 | 1.57 | 65.68 | 20.00 | 0.30 | 53.02 | 14.00 | -0.50 | 45.01 |
| 30.00 | 1.57 | 65.68 | 20.00 | 0.30 | 53.02 | 14.00 | -0.50 | 45.01 |
| 30.00 | 1.57 | 65.68 | 20.00 | 0.30 | 53.02 | 14.00 | -0.50 | 45.01 |
| 30.00 | 1.57 | 65.68 | 20.00 | 0.30 | 53.02 | 14.00 | -0.50 | 45.01 |
| 30.00 | 1.57 | 65.68 | 20.00 | 0.30 | 53.02 | 14.00 | -0.50 | 45.01 |
| 30.00 | 1.57 | 65.68 | 20.00 | 0.30 | 53.02 | 14.00 | -0.50 | 45.01 |
| 30.00 | 1.57 | 65.68 | 20.00 | 0.30 | 53.02 | 14.00 | -0.50 | 45.01 |
| 29.00 | 1.44 | 64.36 | 20.00 | 0.30 | 53.02 | 14.00 | -0.50 | 45.01 |
| 29.00 | 1.44 | 64.36 | 20.00 | 0.30 | 53.02 | 14.00 | -0.50 | 45.01 |
| 29.00 | 1.44 | 64.36 | 20.00 | 0.30 | 53.02 | 14.00 | -0.50 | 45.01 |
| 29.00 | 1.44 | 64.36 | 20.00 | 0.30 | 53.02 | 14.00 | -0.50 | 45.01 |
| 29.00 | 1.44 | 64.36 | 20.00 | 0.30 | 53.02 | 14.00 | -0.50 | 45.01 |
| 29.00 | 1.44 | 64.36 | 20.00 | 0.30 | 53.02 | 14.00 | -0.50 | 45.01 |
| 29.00 | 1.44 | 64.36 | 20.00 | 0.30 | 53.02 | 14.00 | -0.50 | 45.01 |
| 29.00 | 1.44 | 64.36 | 20.00 | 0.30 | 53.02 | 14.00 | -0.50 | 45.01 |
| 29.00 | 1.44 | 64.36 | 20.00 | 0.30 | 53.02 | 14.00 | -0.50 | 45.01 |
| 29.00 | 1.44 | 64.36 | 20.00 | 0.30 | 53.02 | 14.00 | -0.50 | 45.01 |
| 29.00 | 1.44 | 64.36 | 20.00 | 0.30 | 53.02 | 14.00 | -0.50 | 45.01 |
| 29.00 | 1.44 | 64.36 | 20.00 | 0.30 | 53.02 | 14.00 | -0.50 | 45.01 |
| 29.00 | 1.44 | 64.36 | 20.00 | 0.30 | 53.02 | 14.00 | -0.50 | 45.01 |
| 29.00 | 1.44 | 64.36 | 20.00 | 0.30 | 53.02 | 14.00 | -0.50 | 45.01 |
| 29.00 | 1.44 | 64.36 | 20.00 | 0.30 | 53.02 | 14.00 | -0.50 | 45.01 |
| 29.00 | 1.44 | 64.36 | 20.00 | 0.30 | 53.02 | 14.00 | -0.50 | 45.01 |
| 29.00 | 1.44 | 64.36 | 20.00 | 0.30 | 53.02 | 14.00 | -0.50 | 45.01 |
| 29.00 | 1.44 | 64.36 | 20.00 | 0.30 | 53.02 | 14.00 | -0.50 | 45.01 |
| 29.00 | 1.44 | 64.36 | 20.00 | 0.30 | 53.02 | 14.00 | -0.50 | 45.01 |
| 29.00 | 1.44 | 64.36 | 20.00 | 0.30 | 53.02 | 14.00 | -0.50 | 45.01 |
| 29.00 | 1.44 | 64.36 | 20.00 | 0.30 | 53.02 | 14.00 | -0.50 | 45.01 |
| 29.00 | 1.44 | 64.36 | 20.00 | 0.30 | 53.02 | 14.00 | -0.50 | 45.01 |
| 29.00 | 1.44 | 64.36 | 20.00 | 0.30 | 53.02 | 14.00 | -0.50 | 45.01 |
| 29.00 | 1.44 | 64.36 | 20.00 | 0.30 | 53.02 | 14.00 | -0.50 | 45.01 |
| 29.00 | 1.44 | 64.36 | 20.00 | 0.30 | 53.02 | 14.00 | -0.50 | 45.01 |
| 29.00 | 1.44 | 64.36 | 20.00 | 0.30 | 53.02 | 14.00 | -0.50 | 45.01 |
| 29.00 | 1.44 | 64.36 | 20.00 | 0.30 | 53.02 | 14.00 | -0.50 | 45.01 |
| 29.00 | 1.44 | 64.36 | 20.00 | 0.30 | 53.02 | 14.00 | -0.50 | 45.01 |
| 29.00 | 1.44 | 64.36 | 20.00 | 0.30 | 53.02 | 14.00 | -0.50 | 45.01 |
| 29.00 | 1.44 | 64.36 | 20.00 | 0.30 | 53.02 | 14.00 | -0.50 | 45.01 |
| 29.00 | 1.44 | 64.36 | 20.00 | 0.30 | 53.02 | 14.00 | -0.50 | 45.01 |
| 29.00 | 1.44 | 64.36 | 20.00 | 0.30 | 53.02 | 14.00 | -0.50 | 45.01 |
| 29.00 | 1.44 | 64.36 | 20.00 | 0.30 | 53.02 | 14.00 | -0.50 | 45.01 |
| 29.00 | 1.44 | 64.36 | 20.00 | 0.30 | 53.02 | 14.00 | -0.50 | 45.01 |
| 29.00 | 1.44 | 64.36 | 20.00 | 0.30 | 53.02 | 14.00 | -0.50 | 45.01 |
| 29.00 | 1.44 | 64.36 | 20.00 | 0.30 | 53.02 | 14.00 | -0.50 | 45.01 |
| 28.00 | 1.32 | 63.22 | 20.00 | 0.30 | 53.02 | 14.00 | -0.50 | 45.01 |
| 28.00 | 1.32 | 63.22 | 20.00 | 0.30 | 53.02 | 14.00 | -0.50 | 45.01 |
| 28.00 | 1.32 | 63.22 | 20.00 | 0.30 | 53.02 | 14.00 | -0.50 | 45.01 |
| 28.00 | 1.32 | 63.22 | 20.00 | 0.30 | 53.02 | 14.00 | -0.50 | 45.01 |
| 28.00 | 1.32 | 63.22 | 20.00 | 0.30 | 53.02 | 14.00 | -0.50 | 45.01 |
| 28.00 | 1.32 | 63.22 | 20.00 | 0.30 | 53.02 | 14.00 | -0.50 | 45.01 |
| 28.00 | 1.32 | 63.22 | 20.00 | 0.30 | 53.02 | 14.00 | -0.50 | 45.01 |
| 28.00 | 1.32 | 63.22 | 20.00 | 0.30 | 53.02 | 14.00 | -0.50 | 45.01 |
| 28.00 | 1.32 | 63.22 | 20.00 | 0.30 | 53.02 | 14.00 | -0.50 | 45.01 |
| 28.00 | 1.32 | 63.22 | 20.00 | 0.30 | 53.02 | 14.00 | -0.50 | 45.01 |
| 28.00 | 1.32 | 63.22 | 20.00 | 0.30 | 53.02 | 14.00 | -0.50 | 45.01 |
| 28.00 | 1.32 | 63.22 | 20.00 | 0.30 | 53.02 | 14.00 | -0.50 | 45.01 |
| 28.00 | 1.32 | 63.22 | 20.00 | 0.30 | 53.02 | 14.00 | -0.50 | 45.01 |
| 28.00 | 1.32 | 63.22 | 20.00 | 0.30 | 53.02 | 14.00 | -0.50 | 45.01 |
| 28.00 | 1.32 | 63.22 | 20.00 | 0.30 | 53.02 | 14.00 | -0.50 | 45.01 |
| 28.00 | 1.32 | 63.22 | 20.00 | 0.30 | 53.02 | 14.00 | -0.50 | 45.01 |
| 28.00 | 1.32 | 63.22 | 20.00 | 0.30 | 53.02 | 14.00 | -0.50 | 45.01 |
| 28.00 | 1.32 | 63.22 | 20.00 | 0.30 | 53.02 | 14.00 | -0.50 | 45.01 |
| 28.00 | 1.32 | 63.22 | 20.00 | 0.30 | 53.02 | 14.00 | -0.50 | 45.01 |
| 28.00 | 1.32 | 63.22 | 20.00 | 0.30 | 53.02 | 14.00 | -0.50 | 45.01 |
| 28.00 | 1.32 | 63.22 | 20.00 | 0.30 | 53.02 | 14.00 | -0.50 | 45.01 |
| 28.00 | 1.32 | 63.22 | 20.00 | 0.30 | 53.02 | 14.00 | -0.50 | 45.01 |
| 28.00 | 1.32 | 63.22 | 20.00 | 0.30 | 53.02 | 14.00 | -0.50 | 45.01 |
| 28.00 | 1.32 | 63.22 | 20.00 | 0.30 | 53.02 | 14.00 | -0.50 | 45.01 |
| 28.00 | 1.32 | 63.22 | 20.00 | 0.30 | 53.02 | 14.00 | -0.50 | 45.01 |
| 28.00 | 1.32 | 63.22 | 20.00 | 0.30 | 53.02 | 14.00 | -0.50 | 45.01 |
| 28.00 | 1.32 | 63.22 | 20.00 | 0.30 | 53.02 | 14.00 | -0.50 | 45.01 |
| 28.00 | 1.32 | 63.22 | 20.00 | 0.30 | 53.02 | 14.00 | -0.50 | 45.01 |
| 28.00 | 1.32 | 63.22 | 20.00 | 0.30 | 53.02 | 14.00 | -0.50 | 45.01 |
| 28.00 | 1.32 | 63.22 | 20.00 | 0.30 | 53.02 | 14.00 | -0.50 | 45.01 |
| 28.00 | 1.32 | 63.22 | 20.00 | 0.30 | 53.02 | 14.00 | -0.50 | 45.01 |
| 28.00 | 1.32 | 63.22 | 20.00 | 0.30 | 53.02 | 14.00 | -0.50 | 45.01 |
| 28.00 | 1.32 | 63.22 | 20.00 | 0.30 | 53.02 | 14.00 | -0.50 | 45.01 |
| 28.00 | 1.32 | 63.22 | 20.00 | 0.30 | 53.02 | 14.00 | -0.50 | 45.01 |
| 28.00 | 1.32 | 63.22 | 20.00 | 0.30 | 53.02 | 14.00 | -0.50 | 45.01 |
| 28.00 | 1.32 | 63.22 | 20.00 | 0.30 | 53.02 | 14.00 | -0.50 | 45.01 |
| 28.00 | 1.32 | 63.22 | 20.00 | 0.30 | 53.02 | 14.00 | -0.50 | 45.01 |
| 28.00 | 1.32 | 63.22 | 20.00 | 0.30 | 53.02 | 13.00 | -0.66 | 43.35 |
| 28.00 | 1.32 | 63.22 | 20.00 | 0.30 | 53.02 | 13.00 | -0.66 | 43.35 |
| 28.00 | 1.32 | 63.22 | 20.00 | 0.30 | 53.02 | 13.00 | -0.66 | 43.35 |
| 28.00 | 1.32 | 63.22 | 20.00 | 0.30 | 53.02 | 13.00 | -0.66 | 43.35 |
| 28.00 | 1.32 | 63.22 | 20.00 | 0.30 | 53.02 | 13.00 | -0.66 | 43.35 |
| 28.00 | 1.32 | 63.22 | 20.00 | 0.30 | 53.02 | 13.00 | -0.66 | 43.35 |
| 27.00 | 1.20 | 62.05 | 20.00 | 0.30 | 53.02 | 13.00 | -0.66 | 43.35 |
| 27.00 | 1.20 | 62.05 | 20.00 | 0.30 | 53.02 | 13.00 | -0.66 | 43.35 |
| 27.00 | 1.20 | 62.05 | 20.00 | 0.30 | 53.02 | 13.00 | -0.66 | 43.35 |
| 27.00 | 1.20 | 62.05 | 20.00 | 0.30 | 53.02 | 13.00 | -0.66 | 43.35 |
| 27.00 | 1.20 | 62.05 | 20.00 | 0.30 | 53.02 | 13.00 | -0.66 | 43.35 |
| 27.00 | 1.20 | 62.05 | 20.00 | 0.30 | 53.02 | 13.00 | -0.66 | 43.35 |
| 27.00 | 1.20 | 62.05 | 20.00 | 0.30 | 53.02 | 13.00 | -0.66 | 43.35 |
| 27.00 | 1.20 | 62.05 | 20.00 | 0.30 | 53.02 | 13.00 | -0.66 | 43.35 |
| 27.00 | 1.20 | 62.05 | 20.00 | 0.30 | 53.02 | 13.00 | -0.66 | 43.35 |
| 27.00 | 1.20 | 62.05 | 19.00 | 0.16 | 51.60 | 13.00 | -0.66 | 43.35 |
| 27.00 | 1.20 | 62.05 | 19.00 | 0.16 | 51.60 | 13.00 | -0.66 | 43.35 |
| 27.00 | 1.20 | 62.05 | 19.00 | 0.16 | 51.60 | 13.00 | -0.66 | 43.35 |
| 27.00 | 1.20 | 62.05 | 19.00 | 0.16 | 51.60 | 13.00 | -0.66 | 43.35 |
| 27.00 | 1.20 | 62.05 | 19.00 | 0.16 | 51.60 | 13.00 | -0.66 | 43.35 |
| 27.00 | 1.20 | 62.05 | 19.00 | 0.16 | 51.60 | 13.00 | -0.66 | 43.35 |
| 27.00 | 1.20 | 62.05 | 19.00 | 0.16 | 51.60 | 13.00 | -0.66 | 43.35 |
| 27.00 | 1.20 | 62.05 | 19.00 | 0.16 | 51.60 | 13.00 | -0.66 | 43.35 |
| 27.00 | 1.20 | 62.05 | 19.00 | 0.16 | 51.60 | 13.00 | -0.66 | 43.35 |
| 27.00 | 1.20 | 62.05 | 19.00 | 0.16 | 51.60 | 13.00 | -0.66 | 43.35 |
| 27.00 | 1.20 | 62.05 | 19.00 | 0.16 | 51.60 | 13.00 | -0.66 | 43.35 |
| 27.00 | 1.20 | 62.05 | 19.00 | 0.16 | 51.60 | 13.00 | -0.66 | 43.35 |
| 27.00 | 1.20 | 62.05 | 19.00 | 0.16 | 51.60 | 13.00 | -0.66 | 43.35 |
| 27.00 | 1.20 | 62.05 | 19.00 | 0.16 | 51.60 | 13.00 | -0.66 | 43.35 |
| 27.00 | 1.20 | 62.05 | 19.00 | 0.16 | 51.60 | 13.00 | -0.66 | 43.35 |
| 27.00 | 1.20 | 62.05 | 19.00 | 0.16 | 51.60 | 13.00 | -0.66 | 43.35 |
| 27.00 | 1.20 | 62.05 | 19.00 | 0.16 | 51.60 | 13.00 | -0.66 | 43.35 |
| 27.00 | 1.20 | 62.05 | 19.00 | 0.16 | 51.60 | 13.00 | -0.66 | 43.35 |
| 27.00 | 1.20 | 62.05 | 19.00 | 0.16 | 51.60 | 13.00 | -0.66 | 43.35 |
| 27.00 | 1.20 | 62.05 | 19.00 | 0.16 | 51.60 | 13.00 | -0.66 | 43.35 |
| 27.00 | 1.20 | 62.05 | 19.00 | 0.16 | 51.60 | 13.00 | -0.66 | 43.35 |
| 27.00 | 1.20 | 62.05 | 19.00 | 0.16 | 51.60 | 13.00 | -0.66 | 43.35 |
| 27.00 | 1.20 | 62.05 | 19.00 | 0.16 | 51.60 | 13.00 | -0.66 | 43.35 |
| 27.00 | 1.20 | 62.05 | 19.00 | 0.16 | 51.60 | 13.00 | -0.66 | 43.35 |
| 27.00 | 1.20 | 62.05 | 19.00 | 0.16 | 51.60 | 13.00 | -0.66 | 43.35 |
| 27.00 | 1.20 | 62.05 | 19.00 | 0.16 | 51.60 | 13.00 | -0.66 | 43.35 |
| 27.00 | 1.20 | 62.05 | 19.00 | 0.16 | 51.60 | 13.00 | -0.66 | 43.35 |
| 27.00 | 1.20 | 62.05 | 19.00 | 0.16 | 51.60 | 13.00 | -0.66 | 43.35 |
| 27.00 | 1.20 | 62.05 | 19.00 | 0.16 | 51.60 | 13.00 | -0.66 | 43.35 |
| 27.00 | 1.20 | 62.05 | 19.00 | 0.16 | 51.60 | 13.00 | -0.66 | 43.35 |
| 27.00 | 1.20 | 62.05 | 19.00 | 0.16 | 51.60 | 13.00 | -0.66 | 43.35 |
| 27.00 | 1.20 | 62.05 | 19.00 | 0.16 | 51.60 | 13.00 | -0.66 | 43.35 |
| 27.00 | 1.20 | 62.05 | 19.00 | 0.16 | 51.60 | 13.00 | -0.66 | 43.35 |
| 27.00 | 1.20 | 62.05 | 19.00 | 0.16 | 51.60 | 13.00 | -0.66 | 43.35 |
| 27.00 | 1.20 | 62.05 | 19.00 | 0.16 | 51.60 | 13.00 | -0.66 | 43.35 |
| 27.00 | 1.20 | 62.05 | 19.00 | 0.16 | 51.60 | 13.00 | -0.66 | 43.35 |
| 27.00 | 1.20 | 62.05 | 19.00 | 0.16 | 51.60 | 13.00 | -0.66 | 43.35 |
| 27.00 | 1.20 | 62.05 | 19.00 | 0.16 | 51.60 | 13.00 | -0.66 | 43.35 |
| 27.00 | 1.20 | 62.05 | 19.00 | 0.16 | 51.60 | 13.00 | -0.66 | 43.35 |
| 27.00 | 1.20 | 62.05 | 19.00 | 0.16 | 51.60 | 13.00 | -0.66 | 43.35 |
| 27.00 | 1.20 | 62.05 | 19.00 | 0.16 | 51.60 | 13.00 | -0.66 | 43.35 |
| 27.00 | 1.20 | 62.05 | 19.00 | 0.16 | 51.60 | 13.00 | -0.66 | 43.35 |
| 27.00 | 1.20 | 62.05 | 19.00 | 0.16 | 51.60 | 13.00 | -0.66 | 43.35 |
| 27.00 | 1.20 | 62.05 | 19.00 | 0.16 | 51.60 | 13.00 | -0.66 | 43.35 |
| 27.00 | 1.20 | 62.05 | 19.00 | 0.16 | 51.60 | 13.00 | -0.66 | 43.35 |
| 27.00 | 1.20 | 62.05 | 19.00 | 0.16 | 51.60 | 13.00 | -0.66 | 43.35 |
| 27.00 | 1.20 | 62.05 | 19.00 | 0.16 | 51.60 | 13.00 | -0.66 | 43.35 |
| 26.00 | 1.07 | 60.73 | 19.00 | 0.16 | 51.60 | 13.00 | -0.66 | 43.35 |
| 26.00 | 1.07 | 60.73 | 19.00 | 0.16 | 51.60 | 13.00 | -0.66 | 43.35 |
| 26.00 | 1.07 | 60.73 | 19.00 | 0.16 | 51.60 | 13.00 | -0.66 | 43.35 |
| 26.00 | 1.07 | 60.73 | 19.00 | 0.16 | 51.60 | 13.00 | -0.66 | 43.35 |
| 26.00 | 1.07 | 60.73 | 19.00 | 0.16 | 51.60 | 13.00 | -0.66 | 43.35 |
| 26.00 | 1.07 | 60.73 | 19.00 | 0.16 | 51.60 | 13.00 | -0.66 | 43.35 |
| 26.00 | 1.07 | 60.73 | 19.00 | 0.16 | 51.60 | 13.00 | -0.66 | 43.35 |
| 26.00 | 1.07 | 60.73 | 19.00 | 0.16 | 51.60 | 13.00 | -0.66 | 43.35 |
| 26.00 | 1.07 | 60.73 | 19.00 | 0.16 | 51.60 | 13.00 | -0.66 | 43.35 |
| 26.00 | 1.07 | 60.73 | 19.00 | 0.16 | 51.60 | 13.00 | -0.66 | 43.35 |
| 26.00 | 1.07 | 60.73 | 19.00 | 0.16 | 51.60 | 13.00 | -0.66 | 43.35 |
| 26.00 | 1.07 | 60.73 | 19.00 | 0.16 | 51.60 | 13.00 | -0.66 | 43.35 |
| 26.00 | 1.07 | 60.73 | 19.00 | 0.16 | 51.60 | 13.00 | -0.66 | 43.35 |
| 26.00 | 1.07 | 60.73 | 19.00 | 0.16 | 51.60 | 13.00 | -0.66 | 43.35 |
| 26.00 | 1.07 | 60.73 | 19.00 | 0.16 | 51.60 | 13.00 | -0.66 | 43.35 |
| 26.00 | 1.07 | 60.73 | 19.00 | 0.16 | 51.60 | 13.00 | -0.66 | 43.35 |
| 26.00 | 1.07 | 60.73 | 19.00 | 0.16 | 51.60 | 13.00 | -0.66 | 43.35 |
| 26.00 | 1.07 | 60.73 | 19.00 | 0.16 | 51.60 | 13.00 | -0.66 | 43.35 |
| 26.00 | 1.07 | 60.73 | 19.00 | 0.16 | 51.60 | 13.00 | -0.66 | 43.35 |
| 26.00 | 1.07 | 60.73 | 19.00 | 0.16 | 51.60 | 13.00 | -0.66 | 43.35 |
| 26.00 | 1.07 | 60.73 | 19.00 | 0.16 | 51.60 | 13.00 | -0.66 | 43.35 |
| 26.00 | 1.07 | 60.73 | 19.00 | 0.16 | 51.60 | 13.00 | -0.66 | 43.35 |
| 26.00 | 1.07 | 60.73 | 19.00 | 0.16 | 51.60 | 13.00 | -0.66 | 43.35 |
| 26.00 | 1.07 | 60.73 | 19.00 | 0.16 | 51.60 | 13.00 | -0.66 | 43.35 |
| 26.00 | 1.07 | 60.73 | 19.00 | 0.16 | 51.60 | 13.00 | -0.66 | 43.35 |
| 26.00 | 1.07 | 60.73 | 19.00 | 0.16 | 51.60 | 13.00 | -0.66 | 43.35 |
| 26.00 | 1.07 | 60.73 | 19.00 | 0.16 | 51.60 | 13.00 | -0.66 | 43.35 |
| 26.00 | 1.07 | 60.73 | 19.00 | 0.16 | 51.60 | 13.00 | -0.66 | 43.35 |
| 26.00 | 1.07 | 60.73 | 19.00 | 0.16 | 51.60 | 13.00 | -0.66 | 43.35 |
| 26.00 | 1.07 | 60.73 | 19.00 | 0.16 | 51.60 | 13.00 | -0.66 | 43.35 |
| 26.00 | 1.07 | 60.73 | 19.00 | 0.16 | 51.60 | 13.00 | -0.66 | 43.35 |
| 26.00 | 1.07 | 60.73 | 19.00 | 0.16 | 51.60 | 13.00 | -0.66 | 43.35 |
| 26.00 | 1.07 | 60.73 | 19.00 | 0.16 | 51.60 | 13.00 | -0.66 | 43.35 |
| 26.00 | 1.07 | 60.73 | 19.00 | 0.16 | 51.60 | 13.00 | -0.66 | 43.35 |
| 26.00 | 1.07 | 60.73 | 19.00 | 0.16 | 51.60 | 12.00 | -0.82 | 41.80 |
| 26.00 | 1.07 | 60.73 | 19.00 | 0.16 | 51.60 | 12.00 | -0.82 | 41.80 |
| 26.00 | 1.07 | 60.73 | 19.00 | 0.16 | 51.60 | 12.00 | -0.82 | 41.80 |
| 26.00 | 1.07 | 60.73 | 19.00 | 0.16 | 51.60 | 12.00 | -0.82 | 41.80 |
| 26.00 | 1.07 | 60.73 | 19.00 | 0.16 | 51.60 | 12.00 | -0.82 | 41.80 |
| 26.00 | 1.07 | 60.73 | 19.00 | 0.16 | 51.60 | 12.00 | -0.82 | 41.80 |
| 26.00 | 1.07 | 60.73 | 19.00 | 0.16 | 51.60 | 12.00 | -0.82 | 41.80 |
| 26.00 | 1.07 | 60.73 | 19.00 | 0.16 | 51.60 | 12.00 | -0.82 | 41.80 |
| 26.00 | 1.07 | 60.73 | 19.00 | 0.16 | 51.60 | 12.00 | -0.82 | 41.80 |
| 26.00 | 1.07 | 60.73 | 19.00 | 0.16 | 51.60 | 12.00 | -0.82 | 41.80 |
| 26.00 | 1.07 | 60.73 | 19.00 | 0.16 | 51.60 | 12.00 | -0.82 | 41.80 |
| 26.00 | 1.07 | 60.73 | 19.00 | 0.16 | 51.60 | 12.00 | -0.82 | 41.80 |
| 26.00 | 1.07 | 60.73 | 19.00 | 0.16 | 51.60 | 12.00 | -0.82 | 41.80 |
| 26.00 | 1.07 | 60.73 | 19.00 | 0.16 | 51.60 | 12.00 | -0.82 | 41.80 |
| 26.00 | 1.07 | 60.73 | 19.00 | 0.16 | 51.60 | 12.00 | -0.82 | 41.80 |
| 26.00 | 1.07 | 60.73 | 19.00 | 0.16 | 51.60 | 12.00 | -0.82 | 41.80 |
| 26.00 | 1.07 | 60.73 | 19.00 | 0.16 | 51.60 | 12.00 | -0.82 | 41.80 |
| 26.00 | 1.07 | 60.73 | 18.00 | 0.04 | 50.39 | 12.00 | -0.82 | 41.80 |
| 26.00 | 1.07 | 60.73 | 18.00 | 0.04 | 50.39 | 12.00 | -0.82 | 41.80 |
| 26.00 | 1.07 | 60.73 | 18.00 | 0.04 | 50.39 | 12.00 | -0.82 | 41.80 |
| 26.00 | 1.07 | 60.73 | 18.00 | 0.04 | 50.39 | 12.00 | -0.82 | 41.80 |
| 25.00 | 0.96 | 59.60 | 18.00 | 0.04 | 50.39 | 12.00 | -0.82 | 41.80 |
| 25.00 | 0.96 | 59.60 | 18.00 | 0.04 | 50.39 | 12.00 | -0.82 | 41.80 |
| 25.00 | 0.96 | 59.60 | 18.00 | 0.04 | 50.39 | 12.00 | -0.82 | 41.80 |
| 25.00 | 0.96 | 59.60 | 18.00 | 0.04 | 50.39 | 12.00 | -0.82 | 41.80 |
| 25.00 | 0.96 | 59.60 | 18.00 | 0.04 | 50.39 | 12.00 | -0.82 | 41.80 |
| 25.00 | 0.96 | 59.60 | 18.00 | 0.04 | 50.39 | 12.00 | -0.82 | 41.80 |
| 25.00 | 0.96 | 59.60 | 18.00 | 0.04 | 50.39 | 12.00 | -0.82 | 41.80 |
| 25.00 | 0.96 | 59.60 | 18.00 | 0.04 | 50.39 | 12.00 | -0.82 | 41.80 |
| 25.00 | 0.96 | 59.60 | 18.00 | 0.04 | 50.39 | 12.00 | -0.82 | 41.80 |
| 25.00 | 0.96 | 59.60 | 18.00 | 0.04 | 50.39 | 12.00 | -0.82 | 41.80 |
| 25.00 | 0.96 | 59.60 | 18.00 | 0.04 | 50.39 | 12.00 | -0.82 | 41.80 |
| 25.00 | 0.96 | 59.60 | 18.00 | 0.04 | 50.39 | 12.00 | -0.82 | 41.80 |
| 25.00 | 0.96 | 59.60 | 18.00 | 0.04 | 50.39 | 12.00 | -0.82 | 41.80 |
| 25.00 | 0.96 | 59.60 | 18.00 | 0.04 | 50.39 | 12.00 | -0.82 | 41.80 |
| 25.00 | 0.96 | 59.60 | 18.00 | 0.04 | 50.39 | 12.00 | -0.82 | 41.80 |
| 25.00 | 0.96 | 59.60 | 18.00 | 0.04 | 50.39 | 12.00 | -0.82 | 41.80 |
| 25.00 | 0.96 | 59.60 | 18.00 | 0.04 | 50.39 | 12.00 | -0.82 | 41.80 |
| 25.00 | 0.96 | 59.60 | 18.00 | 0.04 | 50.39 | 12.00 | -0.82 | 41.80 |
| 25.00 | 0.96 | 59.60 | 18.00 | 0.04 | 50.39 | 12.00 | -0.82 | 41.80 |
| 25.00 | 0.96 | 59.60 | 18.00 | 0.04 | 50.39 | 12.00 | -0.82 | 41.80 |
| 25.00 | 0.96 | 59.60 | 18.00 | 0.04 | 50.39 | 12.00 | -0.82 | 41.80 |
| 25.00 | 0.96 | 59.60 | 18.00 | 0.04 | 50.39 | 12.00 | -0.82 | 41.80 |
| 25.00 | 0.96 | 59.60 | 18.00 | 0.04 | 50.39 | 12.00 | -0.82 | 41.80 |
| 25.00 | 0.96 | 59.60 | 18.00 | 0.04 | 50.39 | 12.00 | -0.82 | 41.80 |
| 25.00 | 0.96 | 59.60 | 18.00 | 0.04 | 50.39 | 12.00 | -0.82 | 41.80 |
| 25.00 | 0.96 | 59.60 | 18.00 | 0.04 | 50.39 | 12.00 | -0.82 | 41.80 |
| 25.00 | 0.96 | 59.60 | 18.00 | 0.04 | 50.39 | 12.00 | -0.82 | 41.80 |
| 25.00 | 0.96 | 59.60 | 18.00 | 0.04 | 50.39 | 12.00 | -0.82 | 41.80 |
| 25.00 | 0.96 | 59.60 | 18.00 | 0.04 | 50.39 | 12.00 | -0.82 | 41.80 |
| 25.00 | 0.96 | 59.60 | 18.00 | 0.04 | 50.39 | 12.00 | -0.82 | 41.80 |
| 25.00 | 0.96 | 59.60 | 18.00 | 0.04 | 50.39 | 12.00 | -0.82 | 41.80 |
| 25.00 | 0.96 | 59.60 | 18.00 | 0.04 | 50.39 | 12.00 | -0.82 | 41.80 |
| 25.00 | 0.96 | 59.60 | 18.00 | 0.04 | 50.39 | 12.00 | -0.82 | 41.80 |
| 25.00 | 0.96 | 59.60 | 18.00 | 0.04 | 50.39 | 12.00 | -0.82 | 41.80 |
| 25.00 | 0.96 | 59.60 | 18.00 | 0.04 | 50.39 | 12.00 | -0.82 | 41.80 |
| 25.00 | 0.96 | 59.60 | 18.00 | 0.04 | 50.39 | 12.00 | -0.82 | 41.80 |
| 25.00 | 0.96 | 59.60 | 18.00 | 0.04 | 50.39 | 12.00 | -0.82 | 41.80 |
| 25.00 | 0.96 | 59.60 | 18.00 | 0.04 | 50.39 | 12.00 | -0.82 | 41.80 |
| 25.00 | 0.96 | 59.60 | 18.00 | 0.04 | 50.39 | 12.00 | -0.82 | 41.80 |
| 25.00 | 0.96 | 59.60 | 18.00 | 0.04 | 50.39 | 12.00 | -0.82 | 41.80 |
| 25.00 | 0.96 | 59.60 | 18.00 | 0.04 | 50.39 | 12.00 | -0.82 | 41.80 |
| 25.00 | 0.96 | 59.60 | 18.00 | 0.04 | 50.39 | 12.00 | -0.82 | 41.80 |
| 25.00 | 0.96 | 59.60 | 18.00 | 0.04 | 50.39 | 12.00 | -0.82 | 41.80 |
| 25.00 | 0.96 | 59.60 | 18.00 | 0.04 | 50.39 | 12.00 | -0.82 | 41.80 |
| 25.00 | 0.96 | 59.60 | 18.00 | 0.04 | 50.39 | 12.00 | -0.82 | 41.80 |
| 25.00 | 0.96 | 59.60 | 18.00 | 0.04 | 50.39 | 12.00 | -0.82 | 41.80 |
| 25.00 | 0.96 | 59.60 | 18.00 | 0.04 | 50.39 | 12.00 | -0.82 | 41.80 |
| 25.00 | 0.96 | 59.60 | 18.00 | 0.04 | 50.39 | 12.00 | -0.82 | 41.80 |
| 25.00 | 0.96 | 59.60 | 18.00 | 0.04 | 50.39 | 12.00 | -0.82 | 41.80 |
| 25.00 | 0.96 | 59.60 | 18.00 | 0.04 | 50.39 | 12.00 | -0.82 | 41.80 |
| 25.00 | 0.96 | 59.60 | 18.00 | 0.04 | 50.39 | 12.00 | -0.82 | 41.80 |
| 25.00 | 0.96 | 59.60 | 18.00 | 0.04 | 50.39 | 12.00 | -0.82 | 41.80 |
| 25.00 | 0.96 | 59.60 | 18.00 | 0.04 | 50.39 | 12.00 | -0.82 | 41.80 |
| 25.00 | 0.96 | 59.60 | 18.00 | 0.04 | 50.39 | 12.00 | -0.82 | 41.80 |
| 25.00 | 0.96 | 59.60 | 18.00 | 0.04 | 50.39 | 12.00 | -0.82 | 41.80 |
| 25.00 | 0.96 | 59.60 | 18.00 | 0.04 | 50.39 | 12.00 | -0.82 | 41.80 |
| 25.00 | 0.96 | 59.60 | 18.00 | 0.04 | 50.39 | 12.00 | -0.82 | 41.80 |
| 25.00 | 0.96 | 59.60 | 18.00 | 0.04 | 50.39 | 12.00 | -0.82 | 41.80 |
| 25.00 | 0.96 | 59.60 | 18.00 | 0.04 | 50.39 | 12.00 | -0.82 | 41.80 |
| 25.00 | 0.96 | 59.60 | 18.00 | 0.04 | 50.39 | 12.00 | -0.82 | 41.80 |
| 25.00 | 0.96 | 59.60 | 18.00 | 0.04 | 50.39 | 12.00 | -0.82 | 41.80 |
| 25.00 | 0.96 | 59.60 | 18.00 | 0.04 | 50.39 | 12.00 | -0.82 | 41.80 |
| 25.00 | 0.96 | 59.60 | 18.00 | 0.04 | 50.39 | 12.00 | -0.82 | 41.80 |
| 25.00 | 0.96 | 59.60 | 18.00 | 0.04 | 50.39 | 12.00 | -0.82 | 41.80 |
| 25.00 | 0.96 | 59.60 | 18.00 | 0.04 | 50.39 | 12.00 | -0.82 | 41.80 |
| 25.00 | 0.96 | 59.60 | 18.00 | 0.04 | 50.39 | 12.00 | -0.82 | 41.80 |
| 25.00 | 0.96 | 59.60 | 18.00 | 0.04 | 50.39 | 12.00 | -0.82 | 41.80 |
| 25.00 | 0.96 | 59.60 | 18.00 | 0.04 | 50.39 | 12.00 | -0.82 | 41.80 |
| 25.00 | 0.96 | 59.60 | 18.00 | 0.04 | 50.39 | 12.00 | -0.82 | 41.80 |
| 24.00 | 0.83 | 58.34 | 18.00 | 0.04 | 50.39 | 12.00 | -0.82 | 41.80 |
| 24.00 | 0.83 | 58.34 | 18.00 | 0.04 | 50.39 | 12.00 | -0.82 | 41.80 |
| 24.00 | 0.83 | 58.34 | 18.00 | 0.04 | 50.39 | 12.00 | -0.82 | 41.80 |
| 24.00 | 0.83 | 58.34 | 18.00 | 0.04 | 50.39 | 12.00 | -0.82 | 41.80 |
| 24.00 | 0.83 | 58.34 | 18.00 | 0.04 | 50.39 | 12.00 | -0.82 | 41.80 |
| 24.00 | 0.83 | 58.34 | 18.00 | 0.04 | 50.39 | 12.00 | -0.82 | 41.80 |
| 24.00 | 0.83 | 58.34 | 18.00 | 0.04 | 50.39 | 12.00 | -0.82 | 41.80 |
| 24.00 | 0.83 | 58.34 | 18.00 | 0.04 | 50.39 | 12.00 | -0.82 | 41.80 |
| 24.00 | 0.83 | 58.34 | 18.00 | 0.04 | 50.39 | 12.00 | -0.82 | 41.80 |
| 24.00 | 0.83 | 58.34 | 18.00 | 0.04 | 50.39 | 12.00 | -0.82 | 41.80 |
| 24.00 | 0.83 | 58.34 | 18.00 | 0.04 | 50.39 | 12.00 | -0.82 | 41.80 |
| 24.00 | 0.83 | 58.34 | 18.00 | 0.04 | 50.39 | 12.00 | -0.82 | 41.80 |
| 24.00 | 0.83 | 58.34 | 18.00 | 0.04 | 50.39 | 12.00 | -0.82 | 41.80 |
| 24.00 | 0.83 | 58.34 | 18.00 | 0.04 | 50.39 | 12.00 | -0.82 | 41.80 |
| 24.00 | 0.83 | 58.34 | 18.00 | 0.04 | 50.39 | 12.00 | -0.82 | 41.80 |
| 24.00 | 0.83 | 58.34 | 18.00 | 0.04 | 50.39 | 12.00 | -0.82 | 41.80 |
| 24.00 | 0.83 | 58.34 | 18.00 | 0.04 | 50.39 | 12.00 | -0.82 | 41.80 |
| 24.00 | 0.83 | 58.34 | 18.00 | 0.04 | 50.39 | 12.00 | -0.82 | 41.80 |
| 24.00 | 0.83 | 58.34 | 18.00 | 0.04 | 50.39 | 12.00 | -0.82 | 41.80 |
| 24.00 | 0.83 | 58.34 | 18.00 | 0.04 | 50.39 | 12.00 | -0.82 | 41.80 |
| 24.00 | 0.83 | 58.34 | 18.00 | 0.04 | 50.39 | 12.00 | -0.82 | 41.80 |
| 24.00 | 0.83 | 58.34 | 18.00 | 0.04 | 50.39 | 12.00 | -0.82 | 41.80 |
| 24.00 | 0.83 | 58.34 | 18.00 | 0.04 | 50.39 | 12.00 | -0.82 | 41.80 |
| 24.00 | 0.83 | 58.34 | 18.00 | 0.04 | 50.39 | 12.00 | -0.82 | 41.80 |
| 24.00 | 0.83 | 58.34 | 18.00 | 0.04 | 50.39 | 12.00 | -0.82 | 41.80 |
| 24.00 | 0.83 | 58.34 | 18.00 | 0.04 | 50.39 | 12.00 | -0.82 | 41.80 |
| 24.00 | 0.83 | 58.34 | 18.00 | 0.04 | 50.39 | 12.00 | -0.82 | 41.80 |
| 24.00 | 0.83 | 58.34 | 17.00 | -0.08 | 49.17 | 12.00 | -0.82 | 41.80 |
| 24.00 | 0.83 | 58.34 | 17.00 | -0.08 | 49.17 | 12.00 | -0.82 | 41.80 |
| 24.00 | 0.83 | 58.34 | 17.00 | -0.08 | 49.17 | 12.00 | -0.82 | 41.80 |
| 24.00 | 0.83 | 58.34 | 17.00 | -0.08 | 49.17 | 12.00 | -0.82 | 41.80 |
| 24.00 | 0.83 | 58.34 | 17.00 | -0.08 | 49.17 | 12.00 | -0.82 | 41.80 |
| 24.00 | 0.83 | 58.34 | 17.00 | -0.08 | 49.17 | 12.00 | -0.82 | 41.80 |
| 24.00 | 0.83 | 58.34 | 17.00 | -0.08 | 49.17 | 12.00 | -0.82 | 41.80 |
| 24.00 | 0.83 | 58.34 | 17.00 | -0.08 | 49.17 | 12.00 | -0.82 | 41.80 |
| 24.00 | 0.83 | 58.34 | 17.00 | -0.08 | 49.17 | 11.00 | -1.06 | 39.42 |
| 24.00 | 0.83 | 58.34 | 17.00 | -0.08 | 49.17 | 11.00 | -1.06 | 39.42 |
| 24.00 | 0.83 | 58.34 | 17.00 | -0.08 | 49.17 | 11.00 | -1.06 | 39.42 |
| 24.00 | 0.83 | 58.34 | 17.00 | -0.08 | 49.17 | 11.00 | -1.06 | 39.42 |
| 24.00 | 0.83 | 58.34 | 17.00 | -0.08 | 49.17 | 11.00 | -1.06 | 39.42 |
| 24.00 | 0.83 | 58.34 | 17.00 | -0.08 | 49.17 | 11.00 | -1.06 | 39.42 |
| 24.00 | 0.83 | 58.34 | 17.00 | -0.08 | 49.17 | 11.00 | -1.06 | 39.42 |
| 24.00 | 0.83 | 58.34 | 17.00 | -0.08 | 49.17 | 11.00 | -1.06 | 39.42 |
| 24.00 | 0.83 | 58.34 | 17.00 | -0.08 | 49.17 | 11.00 | -1.06 | 39.42 |
| 24.00 | 0.83 | 58.34 | 17.00 | -0.08 | 49.17 | 11.00 | -1.06 | 39.42 |
| 24.00 | 0.83 | 58.34 | 17.00 | -0.08 | 49.17 | 11.00 | -1.06 | 39.42 |
| 24.00 | 0.83 | 58.34 | 17.00 | -0.08 | 49.17 | 11.00 | -1.06 | 39.42 |
| 24.00 | 0.83 | 58.34 | 17.00 | -0.08 | 49.17 | 11.00 | -1.06 | 39.42 |
| 24.00 | 0.83 | 58.34 | 17.00 | -0.08 | 49.17 | 11.00 | -1.06 | 39.42 |
| 24.00 | 0.83 | 58.34 | 17.00 | -0.08 | 49.17 | 11.00 | -1.06 | 39.42 |
| 24.00 | 0.83 | 58.34 | 17.00 | -0.08 | 49.17 | 11.00 | -1.06 | 39.42 |
| 24.00 | 0.83 | 58.34 | 17.00 | -0.08 | 49.17 | 11.00 | -1.06 | 39.42 |
| 24.00 | 0.83 | 58.34 | 17.00 | -0.08 | 49.17 | 11.00 | -1.06 | 39.42 |
| 24.00 | 0.83 | 58.34 | 17.00 | -0.08 | 49.17 | 11.00 | -1.06 | 39.42 |
| 24.00 | 0.83 | 58.34 | 17.00 | -0.08 | 49.17 | 11.00 | -1.06 | 39.42 |
| 24.00 | 0.83 | 58.34 | 17.00 | -0.08 | 49.17 | 11.00 | -1.06 | 39.42 |
| 24.00 | 0.83 | 58.34 | 17.00 | -0.08 | 49.17 | 11.00 | -1.06 | 39.42 |
| 24.00 | 0.83 | 58.34 | 17.00 | -0.08 | 49.17 | 11.00 | -1.06 | 39.42 |
| 24.00 | 0.83 | 58.34 | 17.00 | -0.08 | 49.17 | 11.00 | -1.06 | 39.42 |
| 24.00 | 0.83 | 58.34 | 17.00 | -0.08 | 49.17 | 11.00 | -1.06 | 39.42 |
| 24.00 | 0.83 | 58.34 | 17.00 | -0.08 | 49.17 | 11.00 | -1.06 | 39.42 |
| 24.00 | 0.83 | 58.34 | 17.00 | -0.08 | 49.17 | 11.00 | -1.06 | 39.42 |
| 24.00 | 0.83 | 58.34 | 17.00 | -0.08 | 49.17 | 11.00 | -1.06 | 39.42 |
| 24.00 | 0.83 | 58.34 | 17.00 | -0.08 | 49.17 | 11.00 | -1.06 | 39.42 |
| 24.00 | 0.83 | 58.34 | 17.00 | -0.08 | 49.17 | 11.00 | -1.06 | 39.42 |
| 24.00 | 0.83 | 58.34 | 17.00 | -0.08 | 49.17 | 11.00 | -1.06 | 39.42 |
| 24.00 | 0.83 | 58.34 | 17.00 | -0.08 | 49.17 | 11.00 | -1.06 | 39.42 |
| 24.00 | 0.83 | 58.34 | 17.00 | -0.08 | 49.17 | 11.00 | -1.06 | 39.42 |
| 24.00 | 0.83 | 58.34 | 17.00 | -0.08 | 49.17 | 11.00 | -1.06 | 39.42 |
| 24.00 | 0.83 | 58.34 | 17.00 | -0.08 | 49.17 | 11.00 | -1.06 | 39.42 |
| 24.00 | 0.83 | 58.34 | 17.00 | -0.08 | 49.17 | 11.00 | -1.06 | 39.42 |
| 24.00 | 0.83 | 58.34 | 17.00 | -0.08 | 49.17 | 11.00 | -1.06 | 39.42 |
| 24.00 | 0.83 | 58.34 | 17.00 | -0.08 | 49.17 | 11.00 | -1.06 | 39.42 |
| 23.00 | 0.71 | 57.13 | 17.00 | -0.08 | 49.17 | 11.00 | -1.06 | 39.42 |
| 23.00 | 0.71 | 57.13 | 17.00 | -0.08 | 49.17 | 11.00 | -1.06 | 39.42 |
| 23.00 | 0.71 | 57.13 | 17.00 | -0.08 | 49.17 | 11.00 | -1.06 | 39.42 |
| 23.00 | 0.71 | 57.13 | 17.00 | -0.08 | 49.17 | 11.00 | -1.06 | 39.42 |
| 23.00 | 0.71 | 57.13 | 17.00 | -0.08 | 49.17 | 11.00 | -1.06 | 39.42 |
| 23.00 | 0.71 | 57.13 | 17.00 | -0.08 | 49.17 | 11.00 | -1.06 | 39.42 |
| 23.00 | 0.71 | 57.13 | 17.00 | -0.08 | 49.17 | 11.00 | -1.06 | 39.42 |
| 23.00 | 0.71 | 57.13 | 17.00 | -0.08 | 49.17 | 11.00 | -1.06 | 39.42 |
| 23.00 | 0.71 | 57.13 | 17.00 | -0.08 | 49.17 | 11.00 | -1.06 | 39.42 |
| 23.00 | 0.71 | 57.13 | 17.00 | -0.08 | 49.17 | 11.00 | -1.06 | 39.42 |
| 23.00 | 0.71 | 57.13 | 17.00 | -0.08 | 49.17 | 11.00 | -1.06 | 39.42 |
| 23.00 | 0.71 | 57.13 | 17.00 | -0.08 | 49.17 | 11.00 | -1.06 | 39.42 |
| 23.00 | 0.71 | 57.13 | 17.00 | -0.08 | 49.17 | 11.00 | -1.06 | 39.42 |
| 23.00 | 0.71 | 57.13 | 17.00 | -0.08 | 49.17 | 11.00 | -1.06 | 39.42 |
| 23.00 | 0.71 | 57.13 | 17.00 | -0.08 | 49.17 | 11.00 | -1.06 | 39.42 |
| 23.00 | 0.71 | 57.13 | 17.00 | -0.08 | 49.17 | 11.00 | -1.06 | 39.42 |
| 23.00 | 0.71 | 57.13 | 17.00 | -0.08 | 49.17 | 11.00 | -1.06 | 39.42 |
| 23.00 | 0.71 | 57.13 | 17.00 | -0.08 | 49.17 | 11.00 | -1.06 | 39.42 |
| 23.00 | 0.71 | 57.13 | 17.00 | -0.08 | 49.17 | 11.00 | -1.06 | 39.42 |
| 23.00 | 0.71 | 57.13 | 17.00 | -0.08 | 49.17 | 11.00 | -1.06 | 39.42 |
| 23.00 | 0.71 | 57.13 | 17.00 | -0.08 | 49.17 | 11.00 | -1.06 | 39.42 |
| 23.00 | 0.71 | 57.13 | 17.00 | -0.08 | 49.17 | 11.00 | -1.06 | 39.42 |
| 23.00 | 0.71 | 57.13 | 17.00 | -0.08 | 49.17 | 11.00 | -1.06 | 39.42 |
| 23.00 | 0.71 | 57.13 | 17.00 | -0.08 | 49.17 | 11.00 | -1.06 | 39.42 |
| 23.00 | 0.71 | 57.13 | 17.00 | -0.08 | 49.17 | 11.00 | -1.06 | 39.42 |
| 23.00 | 0.71 | 57.13 | 17.00 | -0.08 | 49.17 | 11.00 | -1.06 | 39.42 |
| 23.00 | 0.71 | 57.13 | 17.00 | -0.08 | 49.17 | 11.00 | -1.06 | 39.42 |
| 23.00 | 0.71 | 57.13 | 17.00 | -0.08 | 49.17 | 11.00 | -1.06 | 39.42 |
| 23.00 | 0.71 | 57.13 | 17.00 | -0.08 | 49.17 | 11.00 | -1.06 | 39.42 |
| 23.00 | 0.71 | 57.13 | 17.00 | -0.08 | 49.17 | 11.00 | -1.06 | 39.42 |
| 23.00 | 0.71 | 57.13 | 17.00 | -0.08 | 49.17 | 11.00 | -1.06 | 39.42 |
| 23.00 | 0.71 | 57.13 | 17.00 | -0.08 | 49.17 | 11.00 | -1.06 | 39.42 |
| 23.00 | 0.71 | 57.13 | 17.00 | -0.08 | 49.17 | 11.00 | -1.06 | 39.42 |
| 23.00 | 0.71 | 57.13 | 17.00 | -0.08 | 49.17 | 11.00 | -1.06 | 39.42 |
| 23.00 | 0.71 | 57.13 | 17.00 | -0.08 | 49.17 | 11.00 | -1.06 | 39.42 |
| 23.00 | 0.71 | 57.13 | 17.00 | -0.08 | 49.17 | 11.00 | -1.06 | 39.42 |
| 23.00 | 0.71 | 57.13 | 17.00 | -0.08 | 49.17 | 11.00 | -1.06 | 39.42 |
| 23.00 | 0.71 | 57.13 | 17.00 | -0.08 | 49.17 | 11.00 | -1.06 | 39.42 |
| 23.00 | 0.71 | 57.13 | 17.00 | -0.08 | 49.17 | 11.00 | -1.06 | 39.42 |
| 23.00 | 0.71 | 57.13 | 17.00 | -0.08 | 49.17 | 11.00 | -1.06 | 39.42 |
| 23.00 | 0.71 | 57.13 | 17.00 | -0.08 | 49.17 | 11.00 | -1.06 | 39.42 |
| 23.00 | 0.71 | 57.13 | 17.00 | -0.08 | 49.17 | 11.00 | -1.06 | 39.42 |
| 23.00 | 0.71 | 57.13 | 17.00 | -0.08 | 49.17 | 11.00 | -1.06 | 39.42 |
| 23.00 | 0.71 | 57.13 | 17.00 | -0.08 | 49.17 | 10.00 | -1.25 | 37.49 |
| 23.00 | 0.71 | 57.13 | 17.00 | -0.08 | 49.17 | 10.00 | -1.25 | 37.49 |
| 23.00 | 0.71 | 57.13 | 17.00 | -0.08 | 49.17 | 10.00 | -1.25 | 37.49 |
| 23.00 | 0.71 | 57.13 | 17.00 | -0.08 | 49.17 | 10.00 | -1.25 | 37.49 |
| 23.00 | 0.71 | 57.13 | 17.00 | -0.08 | 49.17 | 10.00 | -1.25 | 37.49 |
| 23.00 | 0.71 | 57.13 | 17.00 | -0.08 | 49.17 | 10.00 | -1.25 | 37.49 |
| 23.00 | 0.71 | 57.13 | 17.00 | -0.08 | 49.17 | 10.00 | -1.25 | 37.49 |
| 23.00 | 0.71 | 57.13 | 17.00 | -0.08 | 49.17 | 10.00 | -1.25 | 37.49 |
| 23.00 | 0.71 | 57.13 | 17.00 | -0.08 | 49.17 | 10.00 | -1.25 | 37.49 |
| 23.00 | 0.71 | 57.13 | 17.00 | -0.08 | 49.17 | 10.00 | -1.25 | 37.49 |
| 23.00 | 0.71 | 57.13 | 17.00 | -0.08 | 49.17 | 10.00 | -1.25 | 37.49 |
| 23.00 | 0.71 | 57.13 | 17.00 | -0.08 | 49.17 | 10.00 | -1.25 | 37.49 |
| 23.00 | 0.71 | 57.13 | 17.00 | -0.08 | 49.17 | 10.00 | -1.25 | 37.49 |
| 23.00 | 0.71 | 57.13 | 17.00 | -0.08 | 49.17 | 10.00 | -1.25 | 37.49 |
| 23.00 | 0.71 | 57.13 | 17.00 | -0.08 | 49.17 | 10.00 | -1.25 | 37.49 |
| 23.00 | 0.71 | 57.13 | 17.00 | -0.08 | 49.17 | 10.00 | -1.25 | 37.49 |
| 23.00 | 0.71 | 57.13 | 17.00 | -0.08 | 49.17 | 10.00 | -1.25 | 37.49 |
| 23.00 | 0.71 | 57.13 | 17.00 | -0.08 | 49.17 | 10.00 | -1.25 | 37.49 |
| 23.00 | 0.71 | 57.13 | 17.00 | -0.08 | 49.17 | 10.00 | -1.25 | 37.49 |
| 23.00 | 0.71 | 57.13 | 17.00 | -0.08 | 49.17 | 10.00 | -1.25 | 37.49 |
| 23.00 | 0.71 | 57.13 | 17.00 | -0.08 | 49.17 | 10.00 | -1.25 | 37.49 |
| 23.00 | 0.71 | 57.13 | 17.00 | -0.08 | 49.17 | 10.00 | -1.25 | 37.49 |
| 23.00 | 0.71 | 57.13 | 17.00 | -0.08 | 49.17 | 10.00 | -1.25 | 37.49 |
| 23.00 | 0.71 | 57.13 | 17.00 | -0.08 | 49.17 | 10.00 | -1.25 | 37.49 |
| 23.00 | 0.71 | 57.13 | 17.00 | -0.08 | 49.17 | 10.00 | -1.25 | 37.49 |
| 23.00 | 0.71 | 57.13 | 17.00 | -0.08 | 49.17 | 10.00 | -1.25 | 37.49 |
| 23.00 | 0.71 | 57.13 | 17.00 | -0.08 | 49.17 | 10.00 | -1.25 | 37.49 |
| 23.00 | 0.71 | 57.13 | 17.00 | -0.08 | 49.17 | 10.00 | -1.25 | 37.49 |
| 23.00 | 0.71 | 57.13 | 17.00 | -0.08 | 49.17 | 10.00 | -1.25 | 37.49 |
| 23.00 | 0.71 | 57.13 | 17.00 | -0.08 | 49.17 | 10.00 | -1.25 | 37.49 |
| 23.00 | 0.71 | 57.13 | 17.00 | -0.08 | 49.17 | 10.00 | -1.25 | 37.49 |
| 23.00 | 0.71 | 57.13 | 17.00 | -0.08 | 49.17 | 10.00 | -1.25 | 37.49 |
| 23.00 | 0.71 | 57.13 | 17.00 | -0.08 | 49.17 | 10.00 | -1.25 | 37.49 |
| 23.00 | 0.71 | 57.13 | 17.00 | -0.08 | 49.17 | 10.00 | -1.25 | 37.49 |
| 23.00 | 0.71 | 57.13 | 17.00 | -0.08 | 49.17 | 10.00 | -1.25 | 37.49 |
| 23.00 | 0.71 | 57.13 | 16.00 | -0.24 | 47.62 | 10.00 | -1.25 | 37.49 |
| 23.00 | 0.71 | 57.13 | 16.00 | -0.24 | 47.62 | 10.00 | -1.25 | 37.49 |
| 23.00 | 0.71 | 57.13 | 16.00 | -0.24 | 47.62 | 10.00 | -1.25 | 37.49 |
| 23.00 | 0.71 | 57.13 | 16.00 | -0.24 | 47.62 | 10.00 | -1.25 | 37.49 |
| 23.00 | 0.71 | 57.13 | 16.00 | -0.24 | 47.62 | 10.00 | -1.25 | 37.49 |
| 23.00 | 0.71 | 57.13 | 16.00 | -0.24 | 47.62 | 10.00 | -1.25 | 37.49 |
| 23.00 | 0.71 | 57.13 | 16.00 | -0.24 | 47.62 | 10.00 | -1.25 | 37.49 |
| 23.00 | 0.71 | 57.13 | 16.00 | -0.24 | 47.62 | 10.00 | -1.25 | 37.49 |
| 23.00 | 0.71 | 57.13 | 16.00 | -0.24 | 47.62 | 10.00 | -1.25 | 37.49 |
| 23.00 | 0.71 | 57.13 | 16.00 | -0.24 | 47.62 | 10.00 | -1.25 | 37.49 |
| 23.00 | 0.71 | 57.13 | 16.00 | -0.24 | 47.62 | 10.00 | -1.25 | 37.49 |
| 23.00 | 0.71 | 57.13 | 16.00 | -0.24 | 47.62 | 10.00 | -1.25 | 37.49 |
| 23.00 | 0.71 | 57.13 | 16.00 | -0.24 | 47.62 | 10.00 | -1.25 | 37.49 |
| 22.00 | 0.58 | 55.76 | 16.00 | -0.24 | 47.62 | 10.00 | -1.25 | 37.49 |
| 22.00 | 0.58 | 55.76 | 16.00 | -0.24 | 47.62 | 10.00 | -1.25 | 37.49 |
| 22.00 | 0.58 | 55.76 | 16.00 | -0.24 | 47.62 | 10.00 | -1.25 | 37.49 |
| 22.00 | 0.58 | 55.76 | 16.00 | -0.24 | 47.62 | 10.00 | -1.25 | 37.49 |
| 22.00 | 0.58 | 55.76 | 16.00 | -0.24 | 47.62 | 10.00 | -1.25 | 37.49 |
| 22.00 | 0.58 | 55.76 | 16.00 | -0.24 | 47.62 | 10.00 | -1.25 | 37.49 |
| 22.00 | 0.58 | 55.76 | 16.00 | -0.24 | 47.62 | 10.00 | -1.25 | 37.49 |
| 22.00 | 0.58 | 55.76 | 16.00 | -0.24 | 47.62 | 10.00 | -1.25 | 37.49 |
| 22.00 | 0.58 | 55.76 | 16.00 | -0.24 | 47.62 | 10.00 | -1.25 | 37.49 |
| 22.00 | 0.58 | 55.76 | 16.00 | -0.24 | 47.62 | 10.00 | -1.25 | 37.49 |
| 22.00 | 0.58 | 55.76 | 16.00 | -0.24 | 47.62 | 10.00 | -1.25 | 37.49 |
| 22.00 | 0.58 | 55.76 | 16.00 | -0.24 | 47.62 | 10.00 | -1.25 | 37.49 |
| 22.00 | 0.58 | 55.76 | 16.00 | -0.24 | 47.62 | 10.00 | -1.25 | 37.49 |
| 22.00 | 0.58 | 55.76 | 16.00 | -0.24 | 47.62 | 10.00 | -1.25 | 37.49 |
| 22.00 | 0.58 | 55.76 | 16.00 | -0.24 | 47.62 | 10.00 | -1.25 | 37.49 |
| 22.00 | 0.58 | 55.76 | 16.00 | -0.24 | 47.62 | 10.00 | -1.25 | 37.49 |
| 22.00 | 0.58 | 55.76 | 16.00 | -0.24 | 47.62 | 10.00 | -1.25 | 37.49 |
| 22.00 | 0.58 | 55.76 | 16.00 | -0.24 | 47.62 | 10.00 | -1.25 | 37.49 |
| 22.00 | 0.58 | 55.76 | 16.00 | -0.24 | 47.62 | 10.00 | -1.25 | 37.49 |
| 22.00 | 0.58 | 55.76 | 16.00 | -0.24 | 47.62 | 10.00 | -1.25 | 37.49 |
| 22.00 | 0.58 | 55.76 | 16.00 | -0.24 | 47.62 | 10.00 | -1.25 | 37.49 |
| 22.00 | 0.58 | 55.76 | 16.00 | -0.24 | 47.62 | 10.00 | -1.25 | 37.49 |
| 22.00 | 0.58 | 55.76 | 16.00 | -0.24 | 47.62 | 10.00 | -1.25 | 37.49 |
| 22.00 | 0.58 | 55.76 | 16.00 | -0.24 | 47.62 | 10.00 | -1.25 | 37.49 |
| 22.00 | 0.58 | 55.76 | 16.00 | -0.24 | 47.62 | 10.00 | -1.25 | 37.49 |
| 22.00 | 0.58 | 55.76 | 16.00 | -0.24 | 47.62 | 10.00 | -1.25 | 37.49 |
| 22.00 | 0.58 | 55.76 | 16.00 | -0.24 | 47.62 | 10.00 | -1.25 | 37.49 |
| 22.00 | 0.58 | 55.76 | 16.00 | -0.24 | 47.62 | 10.00 | -1.25 | 37.49 |
| 22.00 | 0.58 | 55.76 | 16.00 | -0.24 | 47.62 | 10.00 | -1.25 | 37.49 |
| 22.00 | 0.58 | 55.76 | 16.00 | -0.24 | 47.62 | 10.00 | -1.25 | 37.49 |
| 22.00 | 0.58 | 55.76 | 16.00 | -0.24 | 47.62 | 10.00 | -1.25 | 37.49 |
| 22.00 | 0.58 | 55.76 | 16.00 | -0.24 | 47.62 | 10.00 | -1.25 | 37.49 |
| 22.00 | 0.58 | 55.76 | 16.00 | -0.24 | 47.62 | 10.00 | -1.25 | 37.49 |
| 22.00 | 0.58 | 55.76 | 16.00 | -0.24 | 47.62 | 10.00 | -1.25 | 37.49 |
| 22.00 | 0.58 | 55.76 | 16.00 | -0.24 | 47.62 | 10.00 | -1.25 | 37.49 |
| 22.00 | 0.58 | 55.76 | 16.00 | -0.24 | 47.62 | 10.00 | -1.25 | 37.49 |
| 22.00 | 0.58 | 55.76 | 16.00 | -0.24 | 47.62 | 10.00 | -1.25 | 37.49 |
| 22.00 | 0.58 | 55.76 | 16.00 | -0.24 | 47.62 | 10.00 | -1.25 | 37.49 |
| 22.00 | 0.58 | 55.76 | 16.00 | -0.24 | 47.62 | 10.00 | -1.25 | 37.49 |
| 22.00 | 0.58 | 55.76 | 16.00 | -0.24 | 47.62 | 10.00 | -1.25 | 37.49 |
| 22.00 | 0.58 | 55.76 | 16.00 | -0.24 | 47.62 | 10.00 | -1.25 | 37.49 |
| 22.00 | 0.58 | 55.76 | 16.00 | -0.24 | 47.62 | 10.00 | -1.25 | 37.49 |
| 22.00 | 0.58 | 55.76 | 16.00 | -0.24 | 47.62 | 10.00 | -1.25 | 37.49 |
| 22.00 | 0.58 | 55.76 | 16.00 | -0.24 | 47.62 | 10.00 | -1.25 | 37.49 |
| 22.00 | 0.58 | 55.76 | 16.00 | -0.24 | 47.62 | 10.00 | -1.25 | 37.49 |
| 22.00 | 0.58 | 55.76 | 16.00 | -0.24 | 47.62 | 10.00 | -1.25 | 37.49 |
| 22.00 | 0.58 | 55.76 | 16.00 | -0.24 | 47.62 | 10.00 | -1.25 | 37.49 |
| 22.00 | 0.58 | 55.76 | 16.00 | -0.24 | 47.62 | 10.00 | -1.25 | 37.49 |
| 22.00 | 0.58 | 55.76 | 16.00 | -0.24 | 47.62 | 10.00 | -1.25 | 37.49 |
| 22.00 | 0.58 | 55.76 | 16.00 | -0.24 | 47.62 | 10.00 | -1.25 | 37.49 |
| 22.00 | 0.58 | 55.76 | 16.00 | -0.24 | 47.62 | 10.00 | -1.25 | 37.49 |
| 22.00 | 0.58 | 55.76 | 16.00 | -0.24 | 47.62 | 10.00 | -1.25 | 37.49 |
| 22.00 | 0.58 | 55.76 | 16.00 | -0.24 | 47.62 | 10.00 | -1.25 | 37.49 |
| 22.00 | 0.58 | 55.76 | 16.00 | -0.24 | 47.62 | 10.00 | -1.25 | 37.49 |
| 22.00 | 0.58 | 55.76 | 16.00 | -0.24 | 47.62 | 10.00 | -1.25 | 37.49 |
| 22.00 | 0.58 | 55.76 | 16.00 | -0.24 | 47.62 | 10.00 | -1.25 | 37.49 |
| 22.00 | 0.58 | 55.76 | 16.00 | -0.24 | 47.62 | 10.00 | -1.25 | 37.49 |
| 22.00 | 0.58 | 55.76 | 16.00 | -0.24 | 47.62 | 10.00 | -1.25 | 37.49 |
| 22.00 | 0.58 | 55.76 | 16.00 | -0.24 | 47.62 | 10.00 | -1.25 | 37.49 |
| 22.00 | 0.58 | 55.76 | 16.00 | -0.24 | 47.62 | 10.00 | -1.25 | 37.49 |
| 22.00 | 0.58 | 55.76 | 16.00 | -0.24 | 47.62 | 10.00 | -1.25 | 37.49 |
| 22.00 | 0.58 | 55.76 | 16.00 | -0.24 | 47.62 | 10.00 | -1.25 | 37.49 |
| 22.00 | 0.58 | 55.76 | 16.00 | -0.24 | 47.62 | 10.00 | -1.25 | 37.49 |
| 22.00 | 0.58 | 55.76 | 16.00 | -0.24 | 47.62 | 10.00 | -1.25 | 37.49 |
| 22.00 | 0.58 | 55.76 | 16.00 | -0.24 | 47.62 | 10.00 | -1.25 | 37.49 |
| 22.00 | 0.58 | 55.76 | 16.00 | -0.24 | 47.62 | 10.00 | -1.25 | 37.49 |
| 22.00 | 0.58 | 55.76 | 16.00 | -0.24 | 47.62 | 10.00 | -1.25 | 37.49 |
| 22.00 | 0.58 | 55.76 | 16.00 | -0.24 | 47.62 | 10.00 | -1.25 | 37.49 |
| 22.00 | 0.58 | 55.76 | 16.00 | -0.24 | 47.62 | 10.00 | -1.25 | 37.49 |
| 22.00 | 0.58 | 55.76 | 16.00 | -0.24 | 47.62 | 10.00 | -1.25 | 37.49 |
| 22.00 | 0.58 | 55.76 | 16.00 | -0.24 | 47.62 | 10.00 | -1.25 | 37.49 |
| 22.00 | 0.58 | 55.76 | 16.00 | -0.24 | 47.62 | 10.00 | -1.25 | 37.49 |
| 22.00 | 0.58 | 55.76 | 16.00 | -0.24 | 47.62 |  |  |  |
