# Supplementary material for: Is the Excessive Use of Microblogs an Internet Addiction? Developing a Scale for Assessing the Excessive Use of Microblogs in Chinese College Students
Source: PLoS One. 2014 Nov 18;9(11):e110960. doi: 10.1371/journal.pone.0110960 (PMC4236055; doi:10.1371/journal.pone.0110960)
Supplement: Supporting Information S5 — The overlap of Micro-blog excessive use (MEU) and Internet addiction (IA). (DOCX) [file pone.0110960.s005.docx]

Is the Excessive Use of Microblogs an Internet Addiction? Developing a Scale for Assessing the Excessive Use of Microblogs in Chinese College Students

Juan Hou^a1^, Zhichao Huang^a2^, Hongxia Li^a3^, Mengqiu Liu^4^, Wei Zhang^2^, Ning Ma^2^, Lizhuang Yang^2^, Feng Gu^2^, Ying Liu^4^, Shenghua Jin^3^, Xiaochu Zhang*^2,5^

**Internet addiction**

| standard | n | Group |
| --- | --- | --- |
| 0-19 | 2 |  |
| 20-49 | 109 | You are an average on-line user. You may surf the Web a bit too long at times, but you have control over your usage. |
| 50-79 | 244 | You are experiencing occasional or frequent problems because of the Internet. You should consider their full impact on your life. |
| 80-100 | 26 | Your Internet usage is causing significant problems in your life. You should elevate the impact of the Internet on your life and address the problems directly caused by you Internet usage. |

**Micro-blog Excessive Use**

| standard | n | Group |
| --- | --- | --- |
| 0-19 | 107 | No-excessive use group |
| 20-26 | 107 | Average- excessive use group |
| 27-32 | 82 | Excessive use group |
| >33 | 85 | Significant- excessive use group |

**The overlap of Micro-blog excessive use (MEU) and Internet addiction (IA)**

| MEU & IA | IA | MEU | No MEU nor IA |
| --- | --- | --- | --- |
| 11 | 15 | 74 | 281 |
